# Anti-PD-1 antibody in combination with radiotherapy as first-line therapy for unresectable intrahepatic cholangiocarcinoma

**DOI:** 10.1186/s12916-024-03381-4

**Published:** 2024-04-19

**Authors:** Meiyan Zhu, Meng Jin, Xiao Zhao, Shunli Shen, Yihan Chen, Han Xiao, Guangyan Wei, Qiang He, Bin Li, Zhenwei Peng

**Affiliations:** 1 Department of Radiation Oncology, The First Affiliated Hospital of Sun Yat-Sen University, Guangzhou, 510080 China; 2 Department of Radiation Therapy, The First Hospital of Jilin University, Changchun, 130021 China; 3 Department of Liver Surgery, Center of Hepato-Pancreato-Biliary Surgery, The First Affiliated Hospital of Sun Yat-sen University, Guangzhou, 510080 China; 4 Key Laboratory for Stem Cells and Tissue Engineering, Ministry of Education, Sun Yat-sen University, Guangzhou, 510080 China; 5 Department of Histology and Embryology, Zhongshan School of Medicine, Sun Yat-sen University, Guangzhou, 510080 China; 6 Division of Interventional Ultrasound, The First Affiliated Hospital of Sun Yat-sen University, Guangzhou, 510080 China; 7 Clinical Trials Unit, The First Affiliated Hospital of Sun Yat-sen University, Guangzhou, 510080 China; 8 Institute of Precision Medicine, The First Affiliated Hospital of Sun Yat-sen University, Guangzhou, 510080 China; 9 Cancer Center, The First Affiliated Hospital of Sun Yat-sen University, Guangzhou, 510080 China; 10 Department of Radiation Oncology, Clinical Trials Unit, Institute of Precision Medicine, Cancer Center, The First Affiliated Hospital of Sun Yat-sen University, No.58 Zhongshan Road 2, Yuexiu District, Guangzhou, 510080 Guangdong China

**Keywords:** Intrahepatic cholangiocarcinoma, Radiotherapy, Immunotherapy, Programmed death ligand-1

## Abstract

**Background:**

Unresectable intrahepatic cholangiocarcinoma (iCCA) has a poor prognosis despite treatment with standard combination chemotherapy. We aimed to evaluate the efficacy and safety of radiotherapy in combination with an anti-PD-1 antibody in unresectable iCCA without distant metastases.

**Methods:**

In this phase II study, patients with histopathologically confirmed unresectable primary or postoperative recurrent iCCA without distant metastases were enrolled. Patients received external radiotherapy with a dose of ≥45 Gy (2-2.5 Gy per fraction), followed by anti-PD-1 immunotherapy (camrelizumab 200 mg once, every 3 weeks) initiated within 7 days after completion of radiotherapy as first-line therapy. The primary endpoint was 1-year progression-free survival (PFS) rate. The secondary end points included safety, objective response rate (ORR), disease control rate (DCR), and overall survival (OS).

**Results:**

From December 2019 to March 2021, 36 patients completed radiotherapy and at least one cycle of immunotherapy and were included in efficacy and safety analyses. The median follow-up was 19.0 months (IQR 12.0-24.0), and the one-year PFS rate was 44.4% (95% CI, 30.8-64.0). The median PFS was 12.0 months (95% CI, 7.5-not estimable); the median OS was 22.0 months (95% CI, 15.0-not estimable). The ORR was 61.1% and the DCR was 86.1%. Seventeen of 36 (47.2%) patients experienced treatment-related adverse effects (AEs) of any grade. The most common AE was reactive cutaneous capillary endothelial proliferation (25.0%). Five (13.9%) patients experienced grade ≥3 treatment-related AEs, including decreased lymphocyte (5.6%), bullous dermatitis (2.8%), decreased platelet count (2.8%), and deep-vein thrombosis (2.8%).

**Conclusions:**

External radiotherapy plus camrelizumab, as first-line therapy, met its primary endpoint and showed antitumor activity and low toxicity levels in patients with unresectable iCCA without distant metastases, warranting further investigation.

**Trial registration:**

NCT03898895. Registered 2 April 2019.

**Supplementary Information:**

The online version contains supplementary material available at 10.1186/s12916-024-03381-4.

## Background

Cholangiocarcinoma (CCA) is the second most common hepatic malignancy, and the incidence and mortality rates of CCA, especially the intrahepatic cholangiocarcinoma (iCCA) has increased over the last 10-20 years [1–3]. Surgery remains the cornerstone of cure in the early stages; however, approximately 70% of patients are initially diagnosed with locally advanced or metastatic diseases that are not amenable to resection [4, 5]. Combination chemotherapy with gemcitabine plus cisplatin as the standard first-line treatment in 410 patients with unresectable or recurrent biliary tract cancer (BTC) including 80 iCCA patients resulted in a median progression-free survival (PFS) of 8.0 months, a median overall survival (OS) of 11.7 months and an incidence rate of 70% or more grade 3 or higher adverse events (AEs) [6]. Hence, there remains an urgent need for more effective and safer treatment strategies for patients with unresectable iCCA.

Over the past decade, immunotherapy for unresectable BTC has been investigated but mainly utilized in CCA patients with high microsatellite instability and deficient mismatch [7]. In previously treated patients with advanced BTC, anti-programmed death-1 (PD-1) monotherapy showed moderate efficacy, with a response rate usually lower than 20%, sometimes lower even than 10% [8], while presenting a manageable safety and tolerability profile [9–12]. Moreover, one cohort from a phase I study that evaluated nivolumab in combination with chemotherapy as first-line therapy in 30 patients with unresectable or recurrent BTC including 15 iCCA patients, showed median PFS and OS of 4.2 and 15.4 months, respectively, with 90% of patients experiencing grade 3 or higher treatment-related AEs [13]. Recently, two phase 3, double-blind, global studies of durvalumab or pembrolizumab plus standard chemotherapy with gemcitabine plus cisplatin in patients with unresectable advanced or metastatic BTC demonstrated clinical benefit, with a median OS of 12.8 or 12.7 months and 63% or 70% of grade 3 or higher treatment-related AEs [14, 15]. These phase 3 studies including more than half of iCCA patients, first showed that the addition of immunotherapy to chemotherapy as a first-line therapy significantly improved OS without exacerbating toxicity, providing evidence for the use of immune checkpoint inhibitors in unresectable iCCA. However, the limited survival benefit provided by chemotherapy plus immunotherapy has highlighted the need for immunotherapy in combination with other treatments in this setting.

With advances in radiation techniques, radiotherapy has emerged as an important therapy for inoperable CCA, achieving a median OS of approximately 10 to 13 months [16]. External radiotherapy with or without concurrent fluoropyrimidine was considered an option in National Comprehensive Cancer Network guidelines but without high-level evidence. In addition, radiotherapy is used as an immunostimulatory therapy. Preclinical and clinical data demonstrated that radiotherapy promoted T cell infiltration, increased the number of tumor infiltrating lymphocytes, expanded the T-cell receptor repertoire, and upregulated programmed cell death-ligand 1 (PD-L1) and major histocompatibility complex-I expression on tumor cells [17]. In phase I and II trials, radiotherapy combined with immunotherapy initially demonstrated promising antitumor effects and tolerable toxicity in patients with solid tumors, including 6 patients with CCA [18]. Camrelizumab is a humanized high-affinity immunoglobulin G4 kappa monoclonal antibody approved in China for use in various tumor types. In patients with locally advanced esophageal squamous cell carcinoma, radiotherapy plus camrelizumab is a safe and feasible therapy [19]. However, there is a lack of clinical trial data to evaluate the treatment regimen of radiotherapy combined with camrelizumab in unresectable iCCA. Therefore, we developed a phase II study to evaluate the efficacy and safety of radiotherapy in combination with camrelizumab as first-line therapy for patients with unresectable iCCA without distant metastases. We also explored the correlation between biomarkers and survival, including tumor mutational burden (TMB), microsatellite instability (MSI), and PD-L1.

## Methods

### Study Design and Participants

This study was a single-arm, phase II clinical trial evaluating the anti-tumor activity and safety of radiotherapy combined with camrelizumab for the treatment of unresectable iCCA without distant metastases (NCT03898895). The study protocol (see Additional file 1) was approved by the Ethics Committee of the First Affiliated Hospital of Sun Yat-sen University (No.2019234). The study was conducted in accordance with both the Declarations of Helsinki and Istanbul. Written informed consent was obtained from each subject before initiating any study procedures.

Key inclusion criteria were adult patients aged between 18 and 75 years with an Eastern Cooperative Oncology Group performance status score of 0 or 1, and histopathologically confirmed unresectable primary or postoperative recurrent iCCA without distant metastases. All patients did not previously receive radiotherapy or systemic therapy with at least one measurable lesion based on the Response Evaluation Criteria in Solid Tumors (RECIST) guideline, version 1.1 criteria. All patients had an adequate volume of uninvolved liver > 700 mL, adequate hematologic (absolute neutrophil count ≥ 1.5x10^9^/L, hemoglobin concentration ≥ 90 g/L, platelet count ≥ 100 x10^9^/L), adequate hepatic function (albumin ≥ 28 g/L, total bilirubin < 1.5 times the upper limit of normal (ULN), alanine aminotransferase and aspartate aminotransferase < 5×ULN) and adequate renal function (serum creatine < 1.5×ULN, creatinine clearance rate ≥ 45 ml/min), and a life expectancy of at least 12 weeks were also required. Full inclusion and exclusion criteria are available in the Additional file 2.

### Treatment and Assessments

Intensity modulated radiation therapy techniques with daily cone-beam computed tomography image guidance were used to deliver the prescribed dose. Patients received radiotherapy a dose of ≥45 Gy delivered in 2-2.5 Gy daily fractions. Gross tumor volume (GTV) including primary tumor and positive regional lymph nodes was contoured on planning four-dimensional computed tomography fused with diagnostic contrast-enhanced magnetic resonance imaging. Clinical target volume (CTV) included GTV for both primary and nodal disease with an additional 5-mm marginal expansion. The planning target volume was created from the CTV by adding a margin of 5 mm. The prescribed doses were adjusted according to proximity to organs at risk. Radiotherapy was continued until completion or unacceptable toxicity.

Camrelizumab was given intravenously at a dose of 200 mg of every 3-week cycle within 7 days after the completion of radiotherapy. Treatment was continued until disease progression, unacceptable toxicity, or withdrawal of consent.

The tumor response was evaluated every 6 weeks according to RECIST 1.1 criteria [20], independently by two experienced radiologists with more than 10 years of experience in liver imaging. Any inconsistent assessments were settled after further discussion. Complete response (CR) or partial response (PR) was required to be confirmed on a follow-up assessment more than 4 weeks after an initial evaluation. Considering the possibility of pseudoprogression, patients with the initial judgment of disease progression were treated with an additional 200 mg of camrelizumab, and radiologic examination was performed 4 weeks later to confirm whether it was pseudoprogression or disease progression. AEs were graded according to Common Terminology Criteria for Adverse Events (version 5.0).

Tumor biopsy was performed within 7 days before therapy. The analysis of TMB, MSI, and PD-L1 is described in the Additional file 2.

### Outcomes

The primary end point of this study was the 1-year PFS rate, which was defined as the rate of patients free of progressive disease at 1 year according to RECIST 1.1. PFS was defined as the time from the commencement of radiotherapy to disease progression or death from any cause. The secondary end points included safety, ORR, DCR, and OS. ORR was defined as the proportion of participants with a CR or PR. DCR was defined as the proportion of participants with a CR, PR, or stable disease (SD). OS was defined as the time from the commencement of radiotherapy to death from any cause. Exploratory end points included biomarker analysis of TMB, MSI, and PD-L1.

### Statistical Analysis

The sample size calculation is based on the primary outcome of the 1-year PFS rate. The accrual period will be 2 years and the follow-up period will be 1 year. According to previous studies and observational data from our database, the 1-year PFS rate in patients with unresectable iCCA without distant metastases is approximately 20% after standard chemotherapy with gemcitabine plus cisplatin [21]. We expected that the regimen of radiotherapy combined with camrelizumab would increase 1-year PFS to 40%. A sample size of 32 patients was required to provide 80% power to detect this estimated improvement in one sample log-rank test at a two-sided significance level of 0.05. Given a 10% dropout rate, the total sample size needed to be 36 patients.

Continuous variables are presented as medians and ranges or interquartile ranges, and categorical variables are presented as numbers and percentages. PFS and OS were estimated with the Kaplan-Meier method and compared by the log-rank test for different TMB, PD-L1 and microsatellite status subgroups. A two-sided *P* value less than 0.05 was considered statistically significant. Statistical analyses were performed using R version 4.1.1.

## Results

### Patient Characteristics

**Fig. 1:**
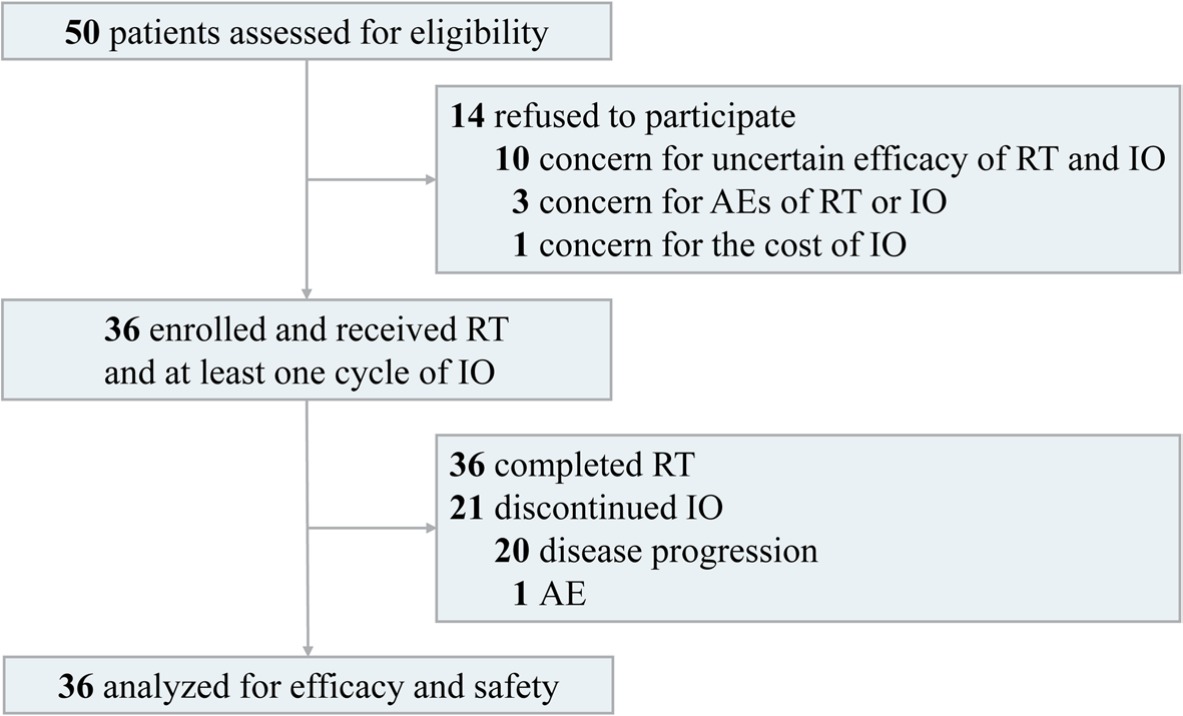
Flow diagram for the phase II study of radiotherapy with anti-PD-1 antibody as first-line therapy treating patients with unresectable iCCA. AE, adverse event; iCCA, intrahepatic cholangiocarcinoma; IO, immunotherapy; PD-1, programmed cell death-1; RT, radiotherapy

Between December 10, 2019, and March 26, 2021, of 50 patients assessed for eligibility, 14 patients refused to participate in the trial, and 36 patients were eligible and included in the efficacy and safety analysis (Fig. 1). Baseline characteristics are summarized in Table 1. More than half of patients were male (63.9%), most had a low TMB status (58.3%), and the median age was 62 years (range, 28-75 years). Nearly 90% of patients had PD-L1-negative status (83.3%) and MSS status (88.9%).

**Table 1:** Baseline patient demographics and disease characteristics

Characteristics	Patients (*N*=36)
**Age, years**	62 (28-75)
**Sex**	
Male	23 (63.9)
Female	13 (36.1)
**ECOG performance status**	
0	28 (77.8)
1	8 (22.2)
**Type of tumor**	
Primary	24 (66.7)
Recurrent	12 (33.3)
**Size of primary tumor, cm**	
≤5	14 (38.9)
>5	22 (61.1)
**Lymph node status**	
Negative	14 (38.9)
Positive	22 (61.1)
**TNM stage**	
II	3 (8.3)
III	33 (91.7)
**CEA, ng/mL**	4 (1-25)
**CA19-9, ng/mL**	1243 (2->12,000)
**TMB status**	
High	15 (41.7)
Low	21 (58.3)
**PD-L1 result**	
Negative	30 (83.3)
Positive	6 (16.7)
**Microsatellite status**	
MSI	4 (11.1)
MSS	32 (88.9)
**Multifocal lesions**	
Single	20 (55.6)
Multiple	16 (44.4)
**Albumin, g/L**	36 (28-49)
**TBIL, μmol/L**	14.3 (3.5-33.0)
**Cirrhosis**	
Yes	11 (30.6)
No	25 (69.4)

Data are median (range) or *n* (%)

*CA* carbohydrate antigen, *CEA* carcinoembryonic antigen, *ECOG* Eastern Cooperative Oncology Group, *MSI* microsatellite instability, *MSS* microsatellite stability, *PD-L1* programmed death-ligand 1, *TBIL* total bilirubin, *TMB* tumor mutational burden, *TNM* tumor-node-metastasis

### Efficacy

**Fig. 2:**
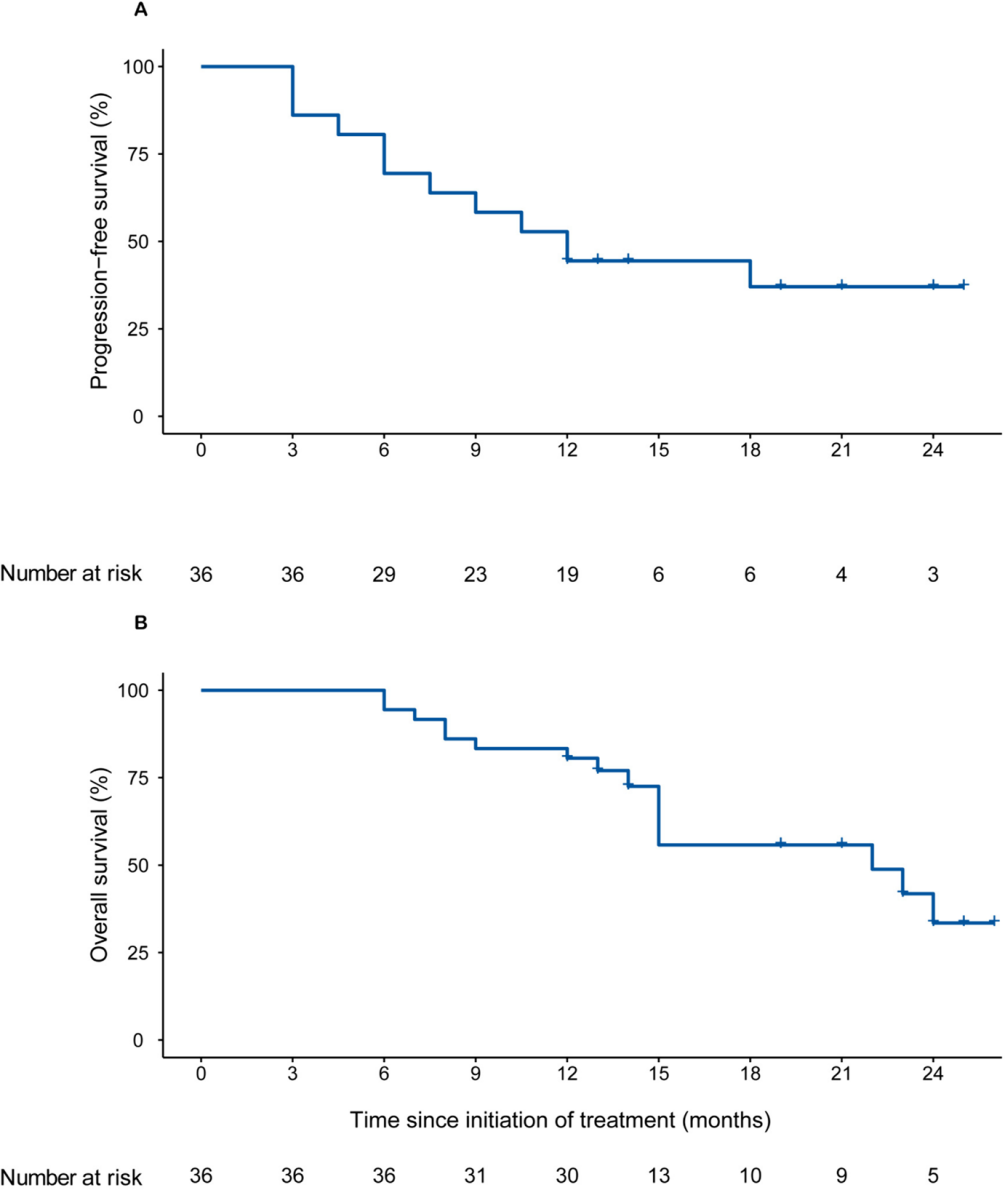
Kaplan-Meier plots of PFS and OS in all included patients. **A** PFS of all 36 enrolled patients. **B** OS of all 36 enrolled patients. OS, overall survival; PFS, progression-free survival

**Fig. 3:**
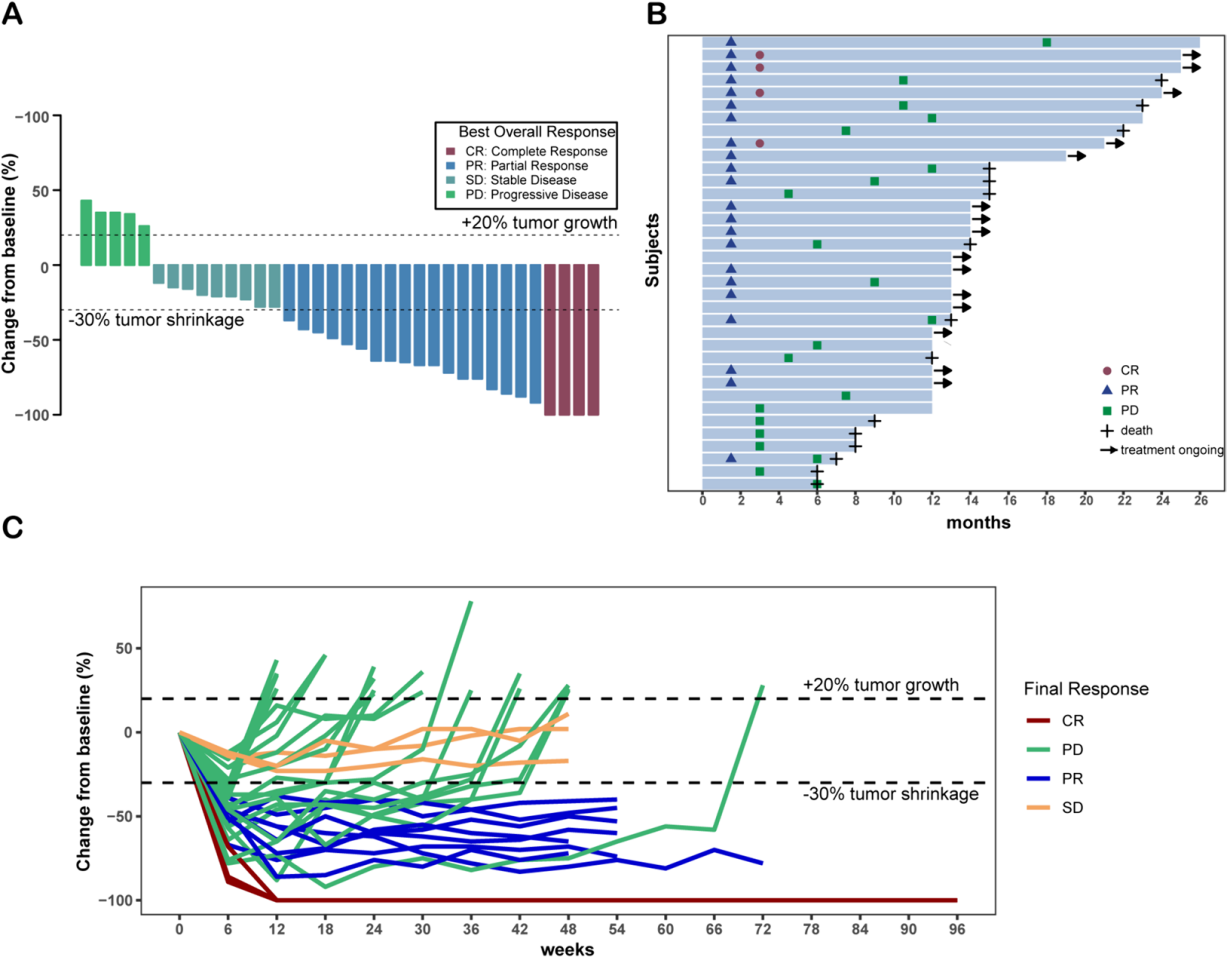
Tumor responses of patients with unresectable iCCA. **A** Waterfall plots of maximal change of tumor size from baseline in target lesion(s) assessed using RECIST version 1.1. **B** Swimmer plot of Percent change in tumor dimension of comparable lesion(s) at best responses. **C** Change of individual tumor burden over time from baseline assessed using RECIST version 1.1. RECIST, Response Evaluation Criteria in Solid Tumors

The median follow-up time was 19.0 months (interquartile ranges, 12.0-24.0 months). All 36 patients completed radiotherapy with a median dose of 50 Gy (range, 45-55 Gy), and patients received camrelizumab with a median cycle number of 15 (range, 2-31) (Additional file 2: Table S1). Fifteen (41.7%) patients were still receiving treatment and 21 (58.3%) patients discontinued treatment, including 20 (55.6%) patients due to progressive disease and one (2.8%) due to AEs. In patients with disease progression, patterns of failure included local, intrahepatic, and distant progression or two of the above. Detailed data are shown in Additional file 2: Table S2. Most progression was intrahepatic combined with distant progression, followed by intrahepatic progression. After discontinuing camrelizumab, 18 patients received chemotherapy and three patients underwent the best supportive care as postprotocol interventions (Additional file 2: Table S3). Twenty-one (58.3%) patients had disease progression or had died, and the median PFS was 12.0 months (95% CI, 7.5-not estimable), with a 1-year PFS rate of 44.4% (95% CI, 30.8-64.0) (Fig. 2A, Table 2). Fifteen (41.7%) deaths occurred, and the median OS was 22.0 months (95% CI, 15.0-not estimable) (Fig. 2B, Table 2). Moreover, 22 (61.1% [95% CI, 43.5-76.9]) patients had an objective response, including 4 (11.1%) CR and 18 (50.0%) PR; 9 (25.0%) patients presented SD and 31 (86.1% [95% CI, 70.5-95.3]) patients had disease control (Fig. 3, Table 2).

**Table 2:** Summary of endpoints: overall response and survival

Variable	Patients (*n*=36)
**Overall response**	
Complete response	4 (11.1)
Partial response	18 (50.0)
Stable disease	9 (25.0)
Progressive disease	5 (13.9)
Objective response rate	61.1 (43.5-76.9)
Disease control rate	86.1 (70.5-95.3)
**One-year progression-free survival rate**	44.4 (30.8-64.0)
**Median overall survival, months**	22.0 (15.0-not estimable)
**Median progression-free survival, months**	12.0 (7.5-not estimable)

Data are *n* (%) or *n* (% [95% CI]), unless specified otherwise. Complete response and partial response were confirmed on a follow-up scan more than 4 weeks after an initial evaluation according to RECIST v1.1

*CI* confidence interval

### Tumor mutational burden, PD-L1 expression, and Microsatellite Instability

**Fig. 4:**
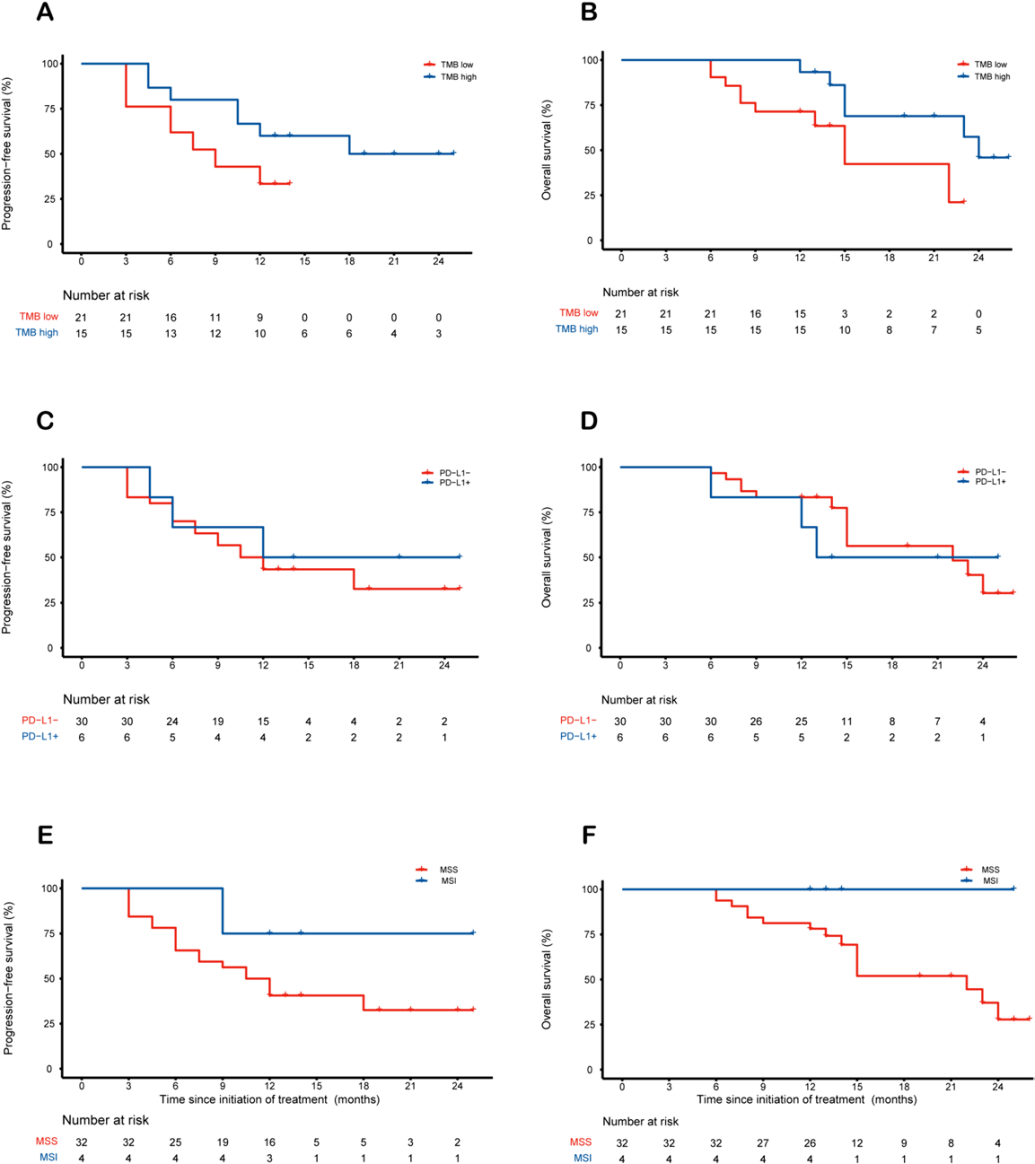
Patient survival in relation to TMB, PD-L1, or microsatellite status and genetic alterations. Kaplan-Meier plots of median (**A**) PFS and (**B**) OS of TMB high versus TMB low patients. Median (**C**) PFS and (**D**) OS of PD-L1 positive versus PD-L1 negative patients. Median (**E**) PFS and (**F**) OS of MSI versus MSS patients. MSI, microsatellite instability; MSS, microsatellite stability; PD-L1, programmed death-ligand 1; TMB, tumor mutational burden

Exploratory subgroup analyses of PFS and OS are shown in Fig. 4. And the results of 1-year PFS, ORR, and DCR in the subgroups of TMB, PD-L1, and microsatellite status were presented in Additional file 2: Table S4. Compared with 21 (58.3%) patients with low TMB, 15 (41.7%) patients with high TMB showed no statistically significant difference in median PFS (18.0 versus 9.0 months, nominal *P* = 0.093). (Figure 4A). And the 1-year PFS rates were 33.3% (95% CI, 18.2-61.0) and 60.0% (95% CI, 39.7-90.7) in the TMB low and high groups, respectively (Additional file 2: Table S4). In addition, median OS was prolonged in patients with high TMB compared with those with low TMB (24.0 versus 15.0 months, nominal *P* = 0.041) (Fig. 4B). Objective response and disease control were achieved in 86.7% and 100% of patients with high TMB compared to 42.9% and 76.2% of patients with low TMB (nominal *P* < 0.001 and nominal *P* = 0.042, respectively). In addition, no obvious difference was found in median PFS (Fig. 4C, E), median OS (Fig. 4D, F), overall response rate (ORR), or disease control rate (DCR) between different PD-L1 and microsatellite status subgroups. However, patients with MSI seemed to have a better trend for ORR and DCR.

### Safety

One patient discontinued treatment because of immune-related bullous dermatitis. Two patients interrupted treatment due to immune-related decreased platelet count or radiotherapy-related deep-vein thrombosis. Seventeen of 36 (47.2%) patients experienced treatment-related AEs of any grade. The most common AEs included reactive cutaneous capillary endothelial proliferation (9 [25.0%]), decreased lymphocyte (8 [22.2%]), decreased white-cell count (8 [22.2%]), and decreased neutrophils (6 [16.7%]). The most common radiotherapy-related AE was decreased lymphocyte (8 [22.2%]), while reactive cutaneous capillary endothelial proliferation (9 [25.0%]) was the most common immune-related AE. Grade 3 or higher AEs occurred in 5 (13.9%) patients, including radiotherapy-related decreased lymphocyte (2 [5.6%]), radiotherapy-related deep-vein thrombosis (1 [2.8%]), immune-related bullous dermatitis (1 [2.8%]), and immune-related decreased platelet count (1 [2.8%]) (Table 3, Additional file 2: Table S5-6). Treatment-related serious AEs occurred in 3 (8.3%) of 36 patients, including radiotherapy-related deep-vein thrombosis (1 [2.8%]), immune-related bullous dermatitis (1 [2.8%]), and immune-related decreased platelet count (1 [2.8%]) (Table 3, Additional file 2: Table S5-6). No unexpected AEs occurred, and no treatment-related deaths occurred.

**Table 3:** Treatment-related adverse events

	Any grade	Grade≥3
Any	17 (47.2)	5 (13.9)
**Hematologic toxic effects**		
Decreased white-cell count	8 (22.2)	0
Decreased lymphocyte	8 (22.2)	2 (5.6)
Decreased neutrophils	6 (16.7)	0
Anemia	2 (5.6)	0
Decreased platelet count	1 (2.8)	1 (2.8)
**Liver function**		
Increased ALT	4 (11.1)	0
Increased AST	3 (8.3)	0
Hypoalbuminemia	2 (5.6)	0
**Nonhematologic toxic effects**		
Reactive cutaneous capillary endothelial proliferation	9 (25.0)	0
Rash	5 (13.9)	0
Abdominal pain	5 (13.9)	0
Fatigue	5 (13.9)	0
Decreased appetite	5 (13.9)	0
Nausea	4 (11.1)	0
Biliary tract infection	4 (11.1)	0
Fever	4 (11.1)	0
Proteinuria	2 (5.6)	0
Hypothyroidism	2 (5.6)	0
Vomiting	2 (5.6)	0
Diarrhea	2 (5.6)	0
Bullous dermatitis	1 (2.8)	1 (2.8)
Deep-vein thrombosis	1 (2.8)	1 (2.8)

Data are *n* (%). Adverse events were graded according to Common Terminology Criteria for Adverse Events version 5.0

*ALT* alanine aminotransferase, *AST* aspartate aminotransferase

## Discussion

To the best of our knowledge, this phase II study is the first to report the safety and efficacy of radiotherapy in combination with an anti-PD-1 antibody as first-line therapy in unresectable iCCA without distant metastases. Our study provides preliminary evidence that radiotherapy combined with the subsequent anti-PD-1 antibody resulted in acceptable antitumor activity and toxicity in patients with unresectable iCCA.

Although radiotherapy is recommended as adjuvant therapy, it is also used in clinical practice for patients with unresectable iCCA. A recent meta-analysis calculated a pooled weighted mean OS of 18.9 months in iCCA patients given external beam radiotherapy, 72.8% of whom received concomitant systemic chemotherapy [22]. The results are heterogeneous ranging from 14.2 to 23.5 months with wide CIs, probably due to the heterogeneity of the radiotherapy modalities and study populations. Preclinical data have demonstrated that radiotherapy can sensitize refractory tumors to PD-1/PD-L1 blockade by modulating the immunogenicity of tumor cells, enhancing antigen-specific CD8+ T-cell responses, and increasing PD-L1 expression on tumor cells and immune cells in the tumor microenvironment [17]. As reported in previous studies, radiotherapy combined with immunotherapy showed antitumor activity in different types of cancer [19, 23–25]. Based on these rationales, radiotherapy combined with immunotherapy strategy is being explored in patients with unresectable iCCA. We recently reported the results of a prospective observational study of patients with unresectable BTC without distant metastases including over 80% CCA treated with radiotherapy plus camrelizumab or chemotherapy. The median disease-free survival and OS in the chemotherapy group were 7.9 and 11.5 months, while the median disease-free survival and OS in the combined therapy group were 12.5 and 17 months, respectively [26].

In this study of radiotherapy combined with camrelizumab, the median PFS and OS were up to 12.0 months and 22.0 months, respectively; the ORR and DCR for all 36 patients were up to 61.1% and 86.1%, respectively. Furthermore, the ABC-02 study of combination chemotherapy with gemcitabine plus cisplatin as the first-line therapy for unresectable BTC resulted in a DCR of 81.4%, a median PFS of 8.0 months, and a median OS of 11.7 months [6]. Additionally, the TOPAZ-1 trial of durvalumab plus chemotherapy as the first-line setting for unresectable BTC showed a median OS of 12.8 months and a median PFS of 7.2 months [14]. Similarly, the KEYNOTE­966 study also showed that patients with unresectable locally advanced or metastatic disease receiving pembrolizumab plus chemotherapy as first-line therapy achieved a median OS of 12.7 months and a median PFS of 6.5 months. These results are lower than those reported in our study. However, the ABC-02, TOPAZ-1, and KEYNOTE­966 studies included both metastatic and locally advanced diseases, among which more than half of patients had CCA. Patients with unresectable iCCA without distant metastases differ in clinical characteristics and prognosis from those with metastatic disease, and therefore they may be treated with alternative treatment strategies. Recently, Edeline *et al.* [27] collected and analyzed the data of iCCA patients from several prospective clinical trials. They reported a 1-year PFS of 37% under treatment with systemic chemotherapy, which was higher than previous studies found but slightly lower than we found. Their findings were derived from advanced liver-only iCCA treated in the first line, while the present study included patients with unresectable primary or postoperative recurrent iCCA without distant metastases rather than advanced liver-only iCCA.

TMB has been suggested to be correlated with a favorable response and survival to immunotherapy in various cancers [12, 28–30]. Consistent with previous findings, in the present study, both response and OS were significantly better in the high TMB group than in the low TMB group. In the KEYNOTE-158 study on pembrolizumab as the second-line or later-line therapy for patients with unresectable BTC, PD-L1-positive patients had an objective response of 6.6%, median PFS of 1.9 months, and median OS of 7.2 months, compared with 2.9%, median PFS of 2.1 months, and median OS of 9.3 months for PD-L1-negative patients [9]. In the TOPAZ-1 study, there was no significant difference in OS and PFS between patients with PD-L1 tumor area positivity of 1% or higher and those with a PD-L1 tumor area positivity score of less than 1% [14]. Similarly, no association between PD-L1 status and clinical efficacy was found in this study. The predictive role of PD-L1 status on efficacy in unresectable iCCA is unclear. Finally, MSI is a predictor for anti-PD-1 treatment in several types of cancers [31]. In this study, due to the influence of population heterogeneity, patients had a relatively higher proportion (11.1%) of MSI, which was expected to be sensitive to anti-PD-1 antibodies. Three of four MSI patients achieved CR or PR, but there was no correlation between microsatellite status and survival outcomes. Further studies are needed in more PD-L1-positive or MSI-high iCCA patients.

In this study, the most common AEs were reactive cutaneous capillary endothelial proliferation, decreased white-cell count, and decreased lymphocyte, and the incidence of grade 3 or higher AEs was only 13.9%, which was notably lower than the 63-71% in patients receiving standard cisplatin plus gemcitabine chemotherapy with or without immunotherapy [6, 14, 15]. In addition, the incidence of grade 3 or higher AEs was similar to that of BTC patients treated with anti-PD-1 antibody monotherapy [11], which suggests that radiotherapy did not increase the toxicity of anti-PD-1 antibody monotherapy. Furthermore, the corresponding incidence rate of grade 3 or higher AEs in our study was generally comparable with those reported in patients with other advanced solid tumors undergoing radiotherapy plus immunotherapy [25].

Transarterial chemoembolization, selective internal radiation therapy, and hepatic arterial infusion chemotherapy, as well as other locoregional treatments, are also reported in iCCA. In the phase II clinical trials, the median PFS and median OS reached 11.8 months and 25.0 months in unresectable iCCA patients receiving hepatic arterial infusion of floxuridine combined with systemic gemcitabine and oxaliplatin, while the corresponding values were 14 months and 22 months among those with combination chemotherapy and selective internal radiation therapy [32, 33]. Another randomized phase II study demonstrated an improved median PFS and median OS in unresectable iCCA treated by system chemotherapy with irinotecan drug-eluting beads therapy by transarterial infusion compared with chemotherapy alone [34]. However, grade 3 or higher AEs were non-negligible in the previous reports and were higher than those of the present strategy. Thus, we will explore better schemes that might be more suitable for different populations based on current and previous findings in future studies.

There are several limitations to this study. First, the sample size of this study was small and patients were enrolled in one center. Furthermore, this study lacked a control group. Second, our statistical assumptions were mainly based on the ABC-02 trial, which included a combination of locally-advanced and metastatic BTC from all sites. Heterogeneity of the population probably introduces bias into the results. Third, although correlation between biomarkers and survival was performed, molecular alterations were absent in the present study, which will be taken into consideration in future trials. A large multicenter randomized phase 3 study is needed to confirm the efficacy and safety of external radiotherapy combined with anti-PD-1 antibody in unresectable iCCA and other types of BTC.

## Conclusions

Our trial met its primary endpoint and showed a positive result, that external radiotherapy plus camrelizumab had acceptable antitumor activity and safety profile in patients with unresectable iCCA without distant metastases. The efficacy of this regimen was related to the TMB status but warranted further investigation in phase 3 randomized controlled trials.

### Supplementary Information

**Additional File 1:** The protocol of clinical trial.

**Additional File 2: Table S1-S6. Table S1** - Treatment exposure. **Table S2** - Disease progression. **Table S3** - Post-protocol interventions. **Table S4** - 1-year PFS rates, ORR, DCR for different TMB, PD-L1 expression and microsatellite instability subgroups. **Table S5** - Radiotherapy-related adverse events. **Table S6** - Immune-related adverse events.

## Data Availability

Data generated or analyzed during the study are available from the corresponding author on reasonable request.

## Abbreviations

AEs: Adverse events
BTC: Biliary tract cancer
CCA: Cholangiocarcinoma
CR: Complete response
CTV: Clinical target volume
DCR: Disease control rate
GTV: Gross tumor volume
iCCA: Intrahepatic cholangiocarcinoma
MSI: Microsatellite instability
ORR: Objective response rate
OS: Overall survival
PD-1: Programmed death-1
PD-L1: Programmed cell death-ligand 1
PFS: Progression-free survival
PR: Partial response
RECIST: Response evaluation criteria in solid tumors
SD: Stable disease
TMB: Tumor mutational burden
ULN: Upper limit of normal
