# Supplementary material for: Anti-PD-1 antibody in combination with radiotherapy as first-line therapy for unresectable intrahepatic cholangiocarcinoma
Source: BMC Med. 2024 Apr 19;22:165. doi: 10.1186/s12916-024-03381-4 (PMC11027363; doi:10.1186/s12916-024-03381-4)
Supplement: Supplementary file 1 — Additional File 1: The protocol of clinical trial. [file 12916_2024_3381_MOESM1_ESM.docx]

**CORRECT**

**COmbination of Radiotherapy with anti-PD-1 antibody for unREseCtable inTrahepatic cholangiocarcinoma:**

**a phase II, single-armed trial**

Protocol Version 1.0 19^th^ March 2019

| Sponsor: | The First Affiliated Hospital, Sun Yat-sen University |
| --- | --- |
| Research Ethics Committee: | ICE for Clinical Research and Animal Trials of the First Affiliated Hospital of Sun Yat-sen University |

**GENERAL INFORMATION**

| **Sponsor** | The First Affiliated Hospital, Sun Yat-sen University |
| --- | --- |
|  |  |
| **Chief Investigator**  Prof Ming Kuang | Department of Liver Surgery, Division of Interventional Ultrasound, The First Affiliated Hospital, Sun Yat-sen University  TEL: +86-20-87755766-8214  FAX: +86-20-87766335  Email: [kuangm@mail.sysu.edu.cn](mailto:kuangm@mail.sysu.edu.cn) |
|  |  |
| **Deputy Clinical Coordinator**  Prof Sui Peng | Clinical Trials Unit, Department of Gastroenterology, The First Affiliated Hospital, Sun Yat-sen University  TEL: +86-87755766-8691  FAX: +86-87755766-8692  Email: [pengsui@vip.163.com](mailto:pengsui@vip.163.com) |
|  |  |
| **Clinical Coordinator**  Dr Zhenwei Peng | Clinical Trials Unit, Department of Radiation Therapy, The First Affiliated Hospital, Sun Yat-sen University  TEL: +86-87755766-8649  FAX: +86-87755766-8692  Email: [pzhenw@mail.sysu.edu.cn](mailto:pzhenw@mail.sysu.edu.cn) |
|  | |
| **Trial Statistician**  Ms Qian Zhou | Biostatistics Team, Clinical Trials Unit, The First Affiliated Hospital, Sun Yat-sen University  TEL: +86-87755766-8691  FAX: +86-87755766-8692  Email: zhouqian2017@163.com |
| Mr Bin Li | Biostatistics Team, Clinical Trials Unit, The First Affiliated Hospital, Sun Yat-sen University  TEL: +86-87755766-8691  FAX: +86-87755766-8692  Email: neolibin@126.com |
|  | |
| **Trial Coordinator**  Ms Jie Mei | Clinical Trials Unit, The First Affiliated Hospital, Sun Yat-sen University  TEL: +86-87755766-8691  FAX: +86-87755766-8692  Email: [mmjj0926@163.com](mailto:mmjj0926@163.com) |

**CORRECT Trial Office**

For general queries, supply of trial materials, and collection of data please contact:

CORRECT Trial Office

Clinical Trials Unit

The First Affiliated Hospital

Sun Yat-sen University

58 Zhongshan 2nd Rd, Yuexiu, Guangzhou, Guangdong, China

TEL: 020-87755766-8649

**Registration**

TEL: 020-87755766-8649

8am-5pm GMT+8 Monday to Friday

**Serious Adverse Event Reporting**

TEL: 020-87755766-8649

**CLINICAL QUERIES**

Clinical queries during office hours should be directed to the trials office

**SIGNATRURE PAGE**

CORRECT Trial Protocol version 1.0 version date 19-03-2019

This protocol has been approved by:

| Name: | Professor Ming Kuang | Trial Role: | Chief Investigator |
| --- | --- | --- | --- |
| Signature: | ___________________________ | Date: | DD / MON / YYYY |
|  |  |  |  |
| Name:* | Professor Ming Kuang | Trial Role: * | Sponsor's representative |
| Signature: * | ___________________________ | Date: * | DD / MON / YYYY |

This protocol describes the CORRECT trial and provides information about procedures for patients taking part in the CORRECT trial. The protocol should not be used as a guide for treatment of patients not taking part in the CORRECT trial.

**AMENDMENTS**

The following amendments and/or administrative changes have been made to this protocol since the implementation of the first approved version.

| Amendment number | Date of amendment | Protocol version number | Type of amendment | Summary of amendment |
| --- | --- | --- | --- | --- |
|  |  |  |  |  |
|  |  |  |  |  |
|  |  |  |  |  |

**Trial Synopsis**

| CORRECT | COmbination of Radiotherapy with anti-PD-1 antibody for unREseCtable inTrahepatic cholangiocarcinoma | | | |
| --- | --- | --- | --- | --- |
| Chief Investigator | Professor Ming Kuang | | | |
| Registration No. | NCT03898895 | Sponsor | The First Affiliated Hospital, Sun Yat-sen University | |
| Trial Aim | Investigate the efficacy and safety of radiotherapy combined with anti-PD-1 antibody for unresectable intrahepatic cholangiocarcinoma. | | | |
| Trial Design | Open label, single-arm phase II clinical trial | | | |
| Phase II trial | Primary Endpoint:   - 1-year progression-free survival (PFS) rate | | | Secondary Endpoints:   - Overall survival (OS) - Adverse events (AE) - Objective response rate (ORR) - Disease control rate (DCR) |
| Patient Population and Sample Size | 36 patients with unresectable intrahepatic cholangiocarcinoma. | | | |
| Key Entry Criteria | | | | |
| *Inclusion* | - Age: 18-75 years; - Histopathologically confirmed unresectable primary or postoperative recurrent intrahepatic cholangiocarcinoma without distant metastasis; - No previous radiotherapy or systemic therapy; - Adequate volume of the uninvolved liver (larger than 700 mL); - At least one measurable lesion based on RECIST 1.1 criteria; - ECOG PS 0-1; - Adequate hematologic, hepatic and renal function: ANC ≥ 1.5x10^9/L, Hb ≥ 90g/L, PLT ≥ 100 x10^9/L, albumin ≥ 28g/L, total bilirubin < 1.5×ULN at diagnosis or after biliary drainage, ALT and AST < 5×ULN, BUN and CREA<1.5×ULN, creatinine clearance rate ≥ 45ml/min; - Life expectancy of at least 12 weeks. | | | |
| *Exclusion* | - Have acute or chronic active hepatitis B or C, HBV-DNA＞2000IU/ml or 10^4^ copy/ml; HCV-RNA＞10^3^ copy/ml; both HBsAg and HCV antibody are positive. If the related results become lower than above standards after antiviral treatment, the patients are qualified for enrolment; - Have distal or perihilar cholangiocarcinoma, gallbladder cancer or carcinoma of the ampulla of Vater; have metastasis in extrahepatic distant organs including lung, central nervous system, bone and etc., or extrahepatic lymph node metastasis beyond abdomen; - Have risky bleeding events requiring transfusion, operation or local therapies, continuous medication in the past 3 months; - Have thromboembolism in the past 6 months, including myocardial infarction, unstable angina, stroke or transient ischemic attack, pulmonary embolism, deep vein thrombosis; - Have taken aspirin (＞325mg/day) or other antiplatelet drugs continuously for 10 days or more within 2 weeks before enrolment; - Uncontrollable hypertension, systolic pressure＞140mmHg or diastolic pressure＞90mmHg after best medical care, or history of hypertensive crisis or hypertensive encephalopathy; - Symptomatic congestive heart failure (NYHA class II-IV). Symptomatic or badly-controlled arrhythmia. Congenital long QT syndrome or modified QTc＞500ms upon screening; - Have active autoimmune diseases that require systemic treatment within 2 years before enrolment; - Active tuberculosis, having antituberculosis therapy at present or within 1 year; - Have a known history of prior invasive malignancies within 5 years before enrolment; - Pregnant or breastfeeding women, or expecting to conceive or father children within the projected duration of the trial; - Have other uncontrollable comorbidities; - Infection of HIV, known syphilis requiring treatment; - Allergic to elements of camrelizumab. | | | |
| Trial Treatment & Assessments | | | | |
| Single-armed phase II trial | - The total radiation dose is over 45Gy without damaging organic function. Conventional intensity-modulated radiotherapy or stereotactic body radiation therapy are both allowed. Camrelizumab 200mg intravenously every 3 weeks will be initiated within 7 days after radiotherapy. Patients will receive camrelizumab until clinical or radiographic disease progression, unacceptable toxicity, death or withdrawal. - Tumor response using contrast-enhanced computed tomography or magnetic resonance imaging should be first performed every 6 weeks. | | | |
| Trial Duration | - 24 months recruitment and 12 months follow-up | | | |
| Trials Office Contact Details  020-87755766-8649 | | | | |

Contents

[**1. Background and rationale** 3](#_Toc104280750)

[**1.1 Epidemiology of intrahepatic cholangiocarcinoma** 3](#_Toc104280751)

[**1.2 Current treatment of unresectable intrahepatic cholangiocarcinoma** 3](#_Toc104280752)

[**1.3 Safety and efficacy of EBRT in unresectable intrahepatic cholangiocarcinoma** 4](#_Toc104280753)

[**1.4 Safety and efficacy of anti-PD-1 antibodies in unresectable intrahepatic cholangiocarcinoma** 5](#_Toc104280754)

[**1.5 Safety and efficacy of radiotherapy combined with anti-PD-1 antibodies for unresectable intrahepatic cholangiocarcinoma** 5](#_Toc104280755)

[**2. Objectives and endpoints** 7](#_Toc104280756)

[**2.1 Objectives** 7](#_Toc104280757)

[**2.2 Primary endpoint** 7](#_Toc104280758)

[**2.3 Secondary endpoints** 7](#_Toc104280759)

[**2.4 Exploratory endpoints** 7](#_Toc104280760)

[**3. Design** 9](#_Toc104280761)

[**4 Follow-up** 10](#_Toc104280762)

[**4.1 Timeframe** 10](#_Toc104280763)

[**4.2 Contents** 10](#_Toc104280764)

[**5. Study population** 12](#_Toc104280765)

[**5.1 Number of patients** 12](#_Toc104280766)

[**5.2 Inclusion criteria** 12](#_Toc104280767)

[**5.3 Exclusion criteria** 12](#_Toc104280768)

[**6. Screening and consent** 14](#_Toc104280769)

[**6.1 Screening** 14](#_Toc104280770)

[**6.2 Informed consent** 14](#_Toc104280771)

[**7. Trial entry** 16](#_Toc104280772)

[**8. Treatment details** 17](#_Toc104280773)

[**8.1 Treatment** 17](#_Toc104280774)

[**8.2 Treatment discontinuation** 17](#_Toc104280775)

[**8.3 Patient withdrawal** 18](#_Toc104280776)

[**8.4 Concomitant therapy** 19](#_Toc104280777)

[**9. Adverse event reporting** 20](#_Toc104280778)

[**9.1 Reporting requirements** 20](#_Toc104280779)

[**9.1.1 Adverse events** 20](#_Toc104280780)

[**9.1.2 Serious adverse events** 20](#_Toc104280781)

[**9.1.3 Reporting period** 21](#_Toc104280782)

[**9.2 Reporting procedure** 21](#_Toc104280783)

[**9.2.1 Site** 21](#_Toc104280784)

[**9.2.2 Trials office** 23](#_Toc104280785)

[**9.2.3** **Reporting to the competent authority and main research ethics committee** 23](#_Toc104280786)

[**9.2.4** **Investigators** 24](#_Toc104280787)

[**9.2.5** **Data monitoring committee** 24](#_Toc104280788)

[**9.2.6** **Manufacturer of advance therapy medicinal product** 24](#_Toc104280789)

[**10.** **Data handling and record keeping** 25](#_Toc104280790)

[**10.1 Data collection** 25](#_Toc104280791)

[**10.2 Archiving** 26](#_Toc104280792)

[**11.** **Quality management** 27](#_Toc104280793)

[**11.1** **Site set-up and initiation** 27](#_Toc104280794)

[**11.2 On-site monitoring** 27](#_Toc104280795)

[**11.3 Central monitoring** 28](#_Toc104280796)

[**11.4 Audit and inspection** 28](#_Toc104280797)

[**12.** **End of trial definition** 29](#_Toc104280798)

[**13.** **Statistical considerations** 30](#_Toc104280799)

[**13.1** **Definition of endpoints** 30](#_Toc104280800)

[**13.2 Analysis populations** 30](#_Toc104280801)

[**13.3 Statistical methods** 30](#_Toc104280802)

[**13.4 Planned interim analysis** 30](#_Toc104280803)

[**13.5 Planned final analysis** 31](#_Toc104280804)

[**13.6 Sample size calculation** 31](#_Toc104280805)

[**14.** **Trial organizational structure** 32](#_Toc104280806)

[**14.1 Sponsor** 32](#_Toc104280807)

[**14.2 Coordinating centre** 32](#_Toc104280808)

[**14.3 Trial management group** 32](#_Toc104280809)

[**14.4 Data monitoring committee** 32](#_Toc104280810)

[**14.5 Finance** 33](#_Toc104280811)

[**15.** **Ethical considerations** 34](#_Toc104280812)

[**16.** **Confidentiality and data protection** 35](#_Toc104280813)

[**17.** **Insurance and indemnity** 36](#_Toc104280814)

[**18.** **Publication policy** 37](#_Toc104280815)

[**19. Reference list** 38](#_Toc104280816)

[**Appendix 1 – Diagnosis** 41](#_Toc104280817)

[**Appendix 2 – Response evaluation criteria in solid tumor** 42](#_Toc104280818)

[**Appendix 3 – ECOG performance status** 43](#_Toc104280819)

[**Appendix 4 – Common terminology criteria for adverse event** 44](#_Toc104280820)

[**Appendix 5 – Definition of adverse events** 45](#_Toc104280821)

[**Appendix 6 – WMA declaration of helsinki** 47](#_Toc104280822)

**1. Background and rationale**

**1.1 Epidemiology of intrahepatic cholangiocarcinoma**

Cholangiocarcinoma (CCA) is the second most common hepatic malignancy after hepatocellular carcinoma (HCC), and the overall incidence of CCA, especially the intrahepatic cholangiocarcinoma (iCCA) has increased progressively worldwide over the past four decades (1-3). Multiple studies reported that the incidence of ICC increased by up to 10-fold over a 3-decade period around the turn of the 20th century in Australia, Japan, the USA, the UK, and across Europe (4). Surgery is the first-line and only curative treatment option for resectable iCCA. However, only approximately 30%-40% of biliary tract cancer patients are amenable to surgical resection (5). The median survival is only 3.3 months in patients with unresectable iCCA who only received supportive therapy (6).

**1.2 Current treatment of unresectable intrahepatic cholangiocarcinoma**

Considering the dismal prognosis, the National Comprehensive Cancer Network (NCCN) guideline recommends several treatment options for patients with unresectable iCCA. Current recommended therapies include gemcitabine-cisplatin combination therapy, fluoropyrimidine-based therapy, gemcitabine-based therapy, external beam radiation therapy (EBRT) combined with concurrent fluoropyrimidine, locoregional therapies, immunotherapy and targeted therapy. Among these recommendations, gemcitabine-cisplatin combination therapy is recommended with the highest-level evidence. Many clinical trials support the fluoropyrimidine-based and gemcitabine-based therapies. However, the benefit of EBRT with concurrent fluoropyrimidine, locoregional therapies, immunotherapy and targeted therapy in unresectable iCCA is not defined due to limited clinical trial data.

Valle J et al. conducted a phase III RCT to evaluate the efficacy and safety of gemcitabine-cisplatin combination therapy versus gemcitabine alone for 410 patients with unresectable, recurrent or metastatic biliary tract cancer (7). 80 patients were with iCCA in the study. They reported a median overall survival (OS) of 11.7 months in the combination group and 8.1 months in the gemcitabine-alone group (*P*<0.001). The median progression-free survival (PFS) was 8.0 months in the combination group and 5.0 months in the gemcitabine-alone group (*P*<0.001). In addition, the rate of tumor control among patients in the combination group was significantly increased (81.4% vs. 71.8%, *P* = 0.049). The rate of grade 3 or 4 toxic effects was 68.8% in the combination group. An alternative therapy of combination of gemcitabine and nab-paclitaxel was investigated by Sahai V et al. in 2018 (8). They enrolled 74 patients with advanced or metastatic CCA including 61 iCCA cases. The study demonstrated an OS of 12.4 months, a PFS of 7.7 months and a disease control rate (DCR) of 66%. Adverse events (grade 3 or higher) were reported in 57% of cases.

Due to the limited survival benefit and nonnegligible adverse events of gemcitabine-based chemotherapies, there is a necessity of developing other therapies to relieve the situation. Besides, since the biology of gallbladder cancer, iCCA and extrahepatic cholangiocarcinoma are distinct (5), iCCA should be separately studied in clinical trials.

**1.3 Safety and efficacy of EBRT in unresectable intrahepatic cholangiocarcinoma**

Several retrospective studies have reported the application of EBRT in patients with unresectable iCCA (9-12). The best reported median OS and PFS were both 30 months. The rate of grade 3 or severer adverse events was less than 15%. Regina V et al. performed a phase I trial and concluded six-fraction stereotactic body radiotherapy (SBRT) with a prescription dose of 32.5 (28.2-48.0) Gy was safe for unresectable iCCA (13). To shed further light upon the role of SBRT in unresectable hepatic malignancies, Ashley A et al. conducted a phase II trial involving 23 patients with iCCA, HCC or HCC/iCCA (14). The radiation dose was 55 (40-55) Gy. Unfortunately, 1 iCCA patient died of liver dysfunction and the discontinuation criteria was prompted thereafter. In detail, the patient underwent renal transplantation and took tacrolimus to which the death was attributed. Upon closed, the median OS and time to progression were 13.2 and 24.7 months in patients with iCCA and HCC/iCCA. Regarding other types of EBRT, hypofractionated proton beam radiation therapy (PBT) was investigated in a phase II trial in 2016 (15). The study enrolled 83 patients with unresectable and localized iCCA or HCC, encompassing 39 iCCA cases, treated by 58.0 (15.1-67.5) Gy PBT. The median OS and PFS were 22.5 and 8.4 months in iCCA patients. No grade 4 or 5 adverse events were observed and the rate of grade 3 complications was 7.7%. Given the above, EBRT is probably an effective and safe alternative for unresectable iCCA patients.

**1.4 Safety and efficacy of anti-PD-1 antibodies in unresectable intrahepatic cholangiocarcinoma**

Programmed death 1(PD-1) is one of the immune checkpoint receptors expressed by activated T cells. The interaction between PD-1 and its ligands, PD-L1, can promote a series of changes and cause exhaustion of T cell, which leads to immune escape of tumor cells (16, 17). Antibodies against PD-1 or PD-L1 have been reported to have valid antitumor activity and safety in varied kinds of carcinoma, including melanoma, lung cancer and HCC (18, 19).

Recently, Sarina et al. summarized the clinical trial results of pembrolizumab as a therapy for advanced biliary tract cancer (20). The study achieved a median PFS of 2.0 months and median OS of 7.4 months in 128 patients. Although the OS was short, it was still satisfying since most of the treated patients were at a late treatment stage whose expected survival was about 3 months with best supportive care. The rate of grade 3 or 4 adverse events was lower than 17% (21). Several clinical trials on checkpoint inhibitors treating CCA are still under evaluation, all of which focused on advanced stage patients (22).

**1.5 Safety and efficacy of radiotherapy combined with anti-PD-1 antibodies for unresectable intrahepatic cholangiocarcinoma**

Preclinical animal experiments have confirmed that radiotherapy combined with anti PD-1/PD-L1 antibodies can achieve synergistic enhancement of immune efficiency (23, 24). There has been an increase of studies evaluating radiotherapy combined with anti PD-1/PD-L1 antibodies. The combination therapy has been studied in 73 patients with metastatic solid tumors including 6 CCA and the patients underwent a median of 5 prior therapies (25). Outcomes were as follows: ORR was 13.2%, OS and PFS were 9.6 and 3.1 months. The rate of grade 3 or 4 adverse events was only 8.2%. Besides, there was a low incidence of hepatobiliary toxicity and no dose-limiting toxicity occurred in liver metastases. Previous studies of other tumor types have verified that the incidence of adverse events of combination therapy is acceptable and similar to that of anti PD-1 antibodies monotherapy, and fatal complications are rare (23, 25, 26).

**2. Objectives and endpoints**

**2.1 Objectives**

Investigate the efficacy and safety of radiotherapy combined with anti-PD-1 antibody for unresectable intrahepatic cholangiocarcinoma.

**2.2 Primary endpoint**

**1-year progression-free survival (PFS) rate**: defined as the rate of patients free of progressive disease at one year according to RECIST 1.1. Progression-free survival (PFS) was defined as the time from the commencement of radiotherapy until disease progression or death from any cause, whichever happens first. Patients who withdraw or who are lost to follow-up will be censored at the date of the last adequate tumor assessment. Patients not having an event will be censored at the date of the last adequate tumor assessment. If patients don’t have baseline tumor assessments, they will be censored at the date of the first treatment.

**2.3 Secondary endpoints**

**Overall survival (OS)**: defined as the time from the commencement of radiotherapy until death from any cause. Patients who withdraw or are lost to follow-up or still alive will be censored at the date last known to be alive.

**Adverse events (AE)**: adverse events during the treatment period using Common Terminology Criteria for Adverse Events (CTCAE) (version 5.0).

**Objective response rate (ORR)**: defined as the proportion of participants with a complete response or partial response according to the Response Evaluation Criteria in Solid Tumors (RECIST) criteria 1.1.

**Disease control rate (DCR)**: defined as the proportion of participants with a complete response, partial response, or stable disease according to the RECIST criteria 1.1.

**2.4 Exploratory endpoints**

Antitumor activity assessed by following biomarkers will be explored, but more biomarkers should be tested if necessary.

**T-cell–inflamed gene-expression profile (GEP) analysis:** tumor RNA extracted from pretreatment formalin-fixed paraffin-embedded slides will be analyzed and GEP score was calculated as a weighted sum of normalized expression values for the 18 genes.

**Tumor mutational burden (TMB)**: total DNA was extracted from the snap-frozen tissues. TMB, considered as numbers of mutations per megabase (Mb) of genome examined.

**Expression of PD-L1:** assessed in pretreatment samples by 22C3 immunohistochemistry (IHC) staining. PD-L1 positive status was defined as staining in ≥1% of tumor cells or presence of staining of any intensity in tumor-infiltrating immune cells at any intensity.

**Genomic alterations**: including microsatellite stability status, nucleotide variants, short and long insertions and deletions, copy number variants, and gene fusions were assessed.

**Mismatch repair deficiency (MMR)/ microsatellite instability (MSI)**: DNA will be extracted from slides of formalin-fixed and paraffin-embedded tumor and matched normal samples. The size in bases is determined for each microsatellite locus and tumors are designated as MSI if two or more mononucleotide loci vary in length compared to the germline DNA.

**3. Design**

This is an open label, single-arm phase II clinical trial.

The total radiation dose is over 45Gy without damaging organic function. Conventional intensity-modulated radiotherapy or stereotactic body radiation therapy are both allowed. Camrelizumab 200mg intravenously every 3 weeks will be initiated within 7 days after radiotherapy. Patients will receive camrelizumab until clinical or radiographic disease progression, unacceptable toxicity, death or withdrawal. Considering the possibility of pseudoprogression, patients with initial judgment of disease progression were treated with an additional 200 mg of camrelizumab, and radiologic examination was performed 4 weeks later to confirm whether it was pseudoprogression or disease progression. If it is true, the camrelizumab should be stopped.

A total of 36 patients will be recruited. The expected recruitment duration is 24 months. After the treatment, the patients will be followed up until progression, death, or termination of the study for patient withdrawal.

**4 Follow-up**

**4.1 Timeframe**

Tumor response using contrast-enhanced computed tomography or magnetic resonance imaging should be performed every 6 weeks. Physical examination and hematological tests (complete blood count, liver function, biochemical tests) should be perform each week during radiotherapy, then every 3 weeks since the initiation of camrelizumab.

During each follow-up, patients are allowed to arrange their visits flexibly in a predefined 7-day timeframe. For example, a patient is required to visit on day 10, he or she is free to make the visit between day 7 and day 13.

Each patient should insist on a predefined specific type of imaging during follow-up. For patients contraindicated for MRI imaging due to metal implants or other reasons, CT should be performed continuously during each follow-up.

**4.2 Contents**

Following disorders should be recorded during each follow-up to evaluate the safety of different treatment plans:

- Blood and lymphatic system disorders
- Cardiac disorders
- Ear and labyrinth disorders
- Endocrine disorders
- Eye disorders
- Gastrointestinal disorders
- General disorders and administration site conditions
- Hepatobiliary disorders
- Immune system disorders
- Infections and infestations
- Injury, poisoning, and procedural complications
- Metabolism and nutrition disorders
- Musculoskeletal and connective tissue disorders
- Neoplasms benign, malignant, and unspecified (including cysts and polyps)
- Nervous system disorders
- Psychiatric disorders
- Renal and urinary disorders
- Reproductive system and breast disorders
- Respiratory, thoracic, and mediastinal disorders
- Skin and subcutaneous tissue disorders
- Vascular disorders

**5. Study population**

**5.1 Number of patients**

A total of 36 patients will be recruited in the trial. Patients withdrawn from the trial will not be replaced.

**5.2 Inclusion criteria**

1. Age: 18-75 years;
2. Histopathologically confirmed unresectable primary or postoperative recurrent intrahepatic cholangiocarcinoma without distant metastasis (Appendix 1);
3. No previous radiotherapy or systemic therapy;
4. Adequate volume of the uninvolved liver (larger than 700 mL);
5. At least one measurable lesion based on RECIST 1.1 criteria (Appendix 2);
6. ECOG PS 0-1 (Appendix 3);
7. Adequate hematologic, hepatic and renal function: ANC ≥ 1.5x10^9/L, Hb ≥ 90g/L, PLT ≥ 100 x10^9/L, albumin ≥ 28g/L, total bilirubin < 1.5×ULN at diagnosis or after biliary drainage, ALT and AST < 5×ULN, BUN and CREA<1.5×ULN, creatinine clearance rate ≥ 45ml/min;
8. Life expectancy of at least 12 weeks.

**5.3 Exclusion criteria**

1. Have acute or chronic active hepatitis B or C, HBV-DNA＞2000IU/ml or 10^4^ copy/ml; HCV-RNA＞10^3^ copy/ml; both HBsAg and HCV antibody are positive. If the related results become lower than above standards after anti-viral treatment, the patients are qualified for enrollment;
2. Have distal or perihilar cholangiocarcinoma, gallbladder cancer or carcinoma of the ampulla of Vater; have metastasis in extrahepatic distant organs including lung, central nervous system, bone etc., or extrahepatic lymph node metastasis beyond abdomen;
3. Have risky bleeding events requiring transfusion, operation or local therapies, continuous medication in the past 3 months;
4. Have thromboembolism in the past 6 months, including myocardial infarction, unstable angina, stroke or transient ischemic attack, pulmonary embolism, deep vein thrombosis;
5. Have taken aspirin (＞325mg/day) or other antiplatelet drugs continuously for 10 days or more within 2 weeks before enrolment;
6. Uncontrollable hypertension, systolic pressure＞140mmHg or diastolic pressure＞90mmHg after best medical care, or history of hypertensive crisis or hypertensive encephalopathy;
7. Symptomatic congestive heart failure (NYHA class II-IV). Symptomatic or badly-controlled arrhythmia. Congenital long QT syndrome or modified QTc＞500ms upon screening;
8. Have active autoimmune diseases that require systemic treatment within 2 years before enrolment;
9. Active tuberculosis, having antituberculosis therapy at present or within 1 year;
10. Have a known history of prior invasive malignancies within 5 years before enrolment;
11. Pregnant or breastfeeding women, or expecting to conceive or father children within the projected duration of the trial;
12. Have other uncontrollable comorbidities;
13. Infection of HIV, known syphilis requiring treatment;
14. Allergic to elements of camrelizumab.

**6. Screening and consent**

**6.1 Screening**

Potential patients will be identified via clinic referrals.

Investigators will be expected to maintain a Screening Log of all potential study candidates. This Log will include limited information about the potential candidate (e.g. date of birth and gender), the date and outcome of the screening process (e.g. enrolled into study, reason for ineligibility, or refused to participate).

For patients who appear to meet the criteria for participation in the study, the Investigator will provide patient information sheet to allow them to make an informed decision regarding their participation. If informed consent is given, the investigator will conduct a full screening evaluation to ensure that the patient satisfies all inclusion and exclusion criteria. A patient who gives written informed consent and who satisfies all the inclusion and exclusion criteria may be recruited into the study. Note that assessments conducted as standard of care do not require informed consent and may be provided as screening data.

**6.2 Informed consent**

Patients will be required to give informed consent at registration. It is the responsibility of the investigator (research nurses) to obtain written informed consent for each patient prior to entering the trial or, where relevant, prior to evaluating the patient’s suitability for the study.

The Patient Information Sheet is provided to facilitate this process. Investigators must ensure that they adequately explain the aim, trial treatment, anticipated benefits and potential hazards of taking part in the trial to the patient. The investigator should also stress that the patient is completely free to refuse to take part or withdraw from the trial at any time. The patient should be given ample time (e.g. 24 hours) to read the Patient Information Sheet and to discuss their participation with others outside of the site research team. The patient must be given an opportunity to ask questions which should be answered to their satisfaction. The right of the patient to refuse to participate in the trial without giving a reason must be respected.

If the patient expresses an interest in participating in the trial they should be asked to sign and date the latest version of the Informed Consent Form. The investigator or designate must then sign and date the form. A copy of the Informed Consent Form should be given to the patient, a copy should be filed in the hospital notes, and the original placed in the Investigator Site File (ISF). Once the patient is entered into the trial the patient’s trial number should be entered on the Informed Consent Form maintained in the ISF. In addition, if the patient has given explicit consent, a copy of the signed Informed Consent Form must be sent in the post to the Trials Office for review.

Details of the informed consent discussions should be recorded in the patient’s medical notes, this should include date of, and information regarding, the initial discussion, the date consent was given, with the name of the trial and the version number of the Patient Information Sheet and Informed Consent Form. Throughout the trial the patient should have the opportunity to ask questions about the trial and any new information that may be relevant to the patient’s continued participation should be shared with them in a timely manner. On occasion it may be necessary to re-consent the patient in which case the process above should be followed and the patient’s right to withdraw from the trial respected.

Electronic copies of the Patient Information Sheet and Informed Consent Form are available from the Trials Office and should be printed or photocopied onto the headed paper of the local institution.

Details of all patients approached about the trial should be recorded on the Patient Screening/ Enrolment Log.

**7. Trial entry**

After screening and consent, patient will be registered into the trial. It is recommended that patients commence trial treatment as soon as possible after recruitment, and ideally within 2 weeks. Delays in initiation of treatment longer than this will need to be documented and approved by the CORRECT Trial Office. If a patient discontinues participation in the trial, then their trial number cannot be re-used.

**8. Treatment details**

**8.1 Treatment**

The total radiation dose is over 45Gy without damaging organic function. Conventional intensity-modulated radiotherapy or stereotactic body radiation therapy are both allowed. Camrelizumab 200mg intravenously every 3 weeks will be initiated within 7 days after radiotherapy. Patients will receive camrelizumab until clinical or radiographic disease progression, unacceptable toxicity, death or withdrawal. Considering the possibility of pseudoprogression, patients with initial judgment of disease progression were treated with an additional 200 mg of camrelizumab, and radiologic examination was performed 4 weeks later to confirm whether it was pseudoprogression or disease progression. If progression is confirmed, the camrelizumab should be stopped.

**8.2 Treatment discontinuation**

In the event of discontinuation of trial treatment, full details of the reason(s) for discontinuation should be recorded in the patient’s medical notes and on the appropriate pages on the Case Report Form (CRF). All patients, including non-compliant subjects, should be followed up according to the protocol unless they withdraw consent.

A patient should discontinue the trial drug in the event of any of the following:

• Disease Progression (Radiological disease progression, Clinical disease progression

• Unacceptable toxicity

• Any other adverse event which, in the Investigator’s opinion, requires termination of the trial medication

• Administration of any other anti-tumor therapies during the trial

• Pregnancy

• Any other reason given by the Investigator

• The patient uses illicit drugs or other substances that may, in the opinion of the Investigator, have a reasonable chance of contributing to toxicity or otherwise interfering with results

• The development of a second malignancy that requires treatment

• Request by the patient or a legal representative/relative to stop the treatment

• Death or End of Trial

**8.3 Patient withdrawal**

Participants are free to withdraw from the study at any stage and may be withdrawn by the Investigator at any stage.

The following are justifiable reasons for the Investigator to withdraw a patient from study:

• Unacceptable toxicity

• Unforeseen events: any event which in the judgement of the Investigator makes further treatment inadvisable

• Serious Adverse Event (SAE) requiring discontinuation of treatment

• Serious violation of the study protocol (including persistent patient attendance failure and persistent non-compliance)

• Withdrawal by the Investigator for clinical reasons not related to the study drug treatment

Participant withdrawals will not be replaced. If withdrawal is decided for a patient, we will provide a withdrawal of consent for him/her to choose to withdraw but still provide follow-up data, withdraw but allow data up to the withdrawal day to be used for analysis or withdraw and remove all trial data.

The details of withdrawal should be clearly documented in the patient’s hospital notes and communicated to the Trial Office on a Withdrawal Form.

The following should be clearly documented in the medical notes:

The date and reason the patient withdraws consent. If no reason for withdrawal is specified by the patient concerned this will also need to be documented in the medical notes. The patient should not be pressured in any way to give a reason for withdrawal if he/she does not wish to supply this information.

We will provide counseling service of other therapeutic approaches for patients withdrawing from the study.

**8.4 Concomitant therapy**

All medication that the participant is taking at the time of enrolment will be recorded. Any changes or new medications added during the study will be recorded. The generic drug name, daily dose, route of administration, treatment start/stop date and indication will be recorded.

Participants will be asked to limit alcohol consumption and participants with Alcoholic Liver Disease advised to abstain completely.

Any drug, if considered necessary for the participant, is permitted at the discretion of the Investigator, with the following exceptions: Participation in another trial of an investigational product.

Of note, for patients with hepatitis, anti-viral treatment is allowed during the study. For patients with biliary obstruction, interventional drainage and cholagogue are allowed during the study.

**9. Adverse event reporting**

The collection and reporting of Adverse Events (AEs) will be in accordance with ICH GCP. Definitions of different types of AE are listed in Appendix 5. The Investigator should assess the seriousness and causality (relatedness) of all AEs experienced by the patient (this should be documented in the source data) with reference to the Investigator Brochure.

**9.1 Reporting requirements**

**9.1.1 Adverse events**

All medical occurrences which meet the definition of an AE (see Appendix 5 for definition) should be reported. Please note this includes abnormal laboratory findings.

**9.1.2 Serious adverse events**

Investigators should report AEs that meet the definition of an SAE (see Appendix 5 for definition).

**9.1.2.1 Events that do not require reporting on a Serious Adverse Event Form**

The following events should not be reported on an SAE Form:

• Hospitalizations for:

o Protocol defined treatment

o Pre-planned elective procedures unless the condition worsens

o Treatment for progression of the patient’s cancer

• Progression or death as a result of the patient’s cancer, as this information is captured elsewhere on the Case Report Form

**9.1.2.2 Monitoring pregnancies for potential Serious Adverse Events**

It is important to monitor the outcome of pregnancies of patients in order to provide SAE data on congenital anomalies or birth defects.

In the event that a patient or their partner becomes pregnant during the SAE reporting period please complete a Pregnancy Notification Form (providing the patient’s details) and return to the Trials Office as soon as possible. If it is the patient who is pregnant provide outcome data on a follow-up Pregnancy Notification Form. Where the patient’s partner is pregnant consent must first be obtained and the patient should be given a Release of Medical information form to give to their partner. If the partner is happy to provide information on the outcome of their pregnancy they should sign the Release of Medical information form. Once consent has been obtained provide details of the outcome of the pregnancy on a follow-up Pregnancy Notification Form. If appropriate also complete an SAE Form as detailed below.

**9.1.3 Reporting period**

Details of all AEs (except those listed above) will be documented and reported on entry into the trial until 28 days after the administration of the last treatment.

SAEs that are judged to be at least possibly related to trial treatment must still be reported in an expedited manner irrespective of how long after treatment administration the reaction occurred.

**9.2 Reporting procedure**

**9.2.1 Site**

**9.2.1.1 Adverse events**

AEs should be reported on an AE Form (and where applicable on an SAE Form). An AE Form should be completed at each visit and returned to the CORRECT Trials Office.

AEs will be reviewed using the Common Terminology Criteria for Adverse Events (CTCAE), version 5.0 (see Appendix 4). Any AEs experienced by the patient but not included in the CTCAE should be graded by an Investigator and recorded on the AE Form using a scale of (1) mild, (2) moderate or (3) severe. For each sign/symptom, the highest grade observed since the last visit should be recorded.

**9.2.1.2 Serious adverse events**

For more detailed instructions on SAE reporting refer to the SAE Form Completion Guidelines.

AEs defined as serious and which require reporting as an SAE (excluding events listed in Section 8.1 above) should be reported on an SAE Form. When completing the form, the Investigator will be asked to define the causality and the severity of the AE, which should be documented using the CTCAE version 5.0.

On becoming aware that a patient has experienced an SAE, the Investigator (or delegate) must complete, date and sign an SAE Form. The form should be faxed together with a SAE Fax Cover Sheet to the Trials Office using one of the numbers listed below as soon as possible and no later than 24 hours after first becoming aware of the event:

To report an SAE, fax the SAE Form with an SAE Fax Cover Sheet to:

020-8733 1952

On receipt the Trials Office will allocate each SAE a unique reference number. This number will be transcribed onto the SAE Fax Cover Sheet which will then be faxed back to the site as proof of receipt. If confirmation of receipt is not received within 1 working day please contact the Trials Office. The SAE reference number should be quoted on all correspondence and follow-up reports regarding the SAE. The SAE Fax Cover Sheet completed by the Trials Office should be filed with the SAE Form in the ISF.

For SAE Forms completed by someone other than the Investigator the Investigator will be required to countersign the original SAE Form to confirm agreement with the causality and severity assessments. The form should then be returned to the Trials Office in the post and a copy kept in the ISF.

**9.2.1.3 Provision of follow-up information**

Patients should be followed up until resolution or stabilization of the event. Follow-up information should be provided on a new SAE Form (refer to the SAE Form Completion Guidelines for further information).

**9.2.2 Trials office**

On receipt of an SAE Form seriousness and causality will be determined independently by a Clinical Coordinator. An SAE judged by the Investigator or Clinical Coordinator to have a reasonable causal relationship with the trial medication will be regarded as a Serious Adverse Reaction (SAR). The Clinical Coordinator will also assess all SARs for expectedness. If the event meets the definition of a SAR that is unexpected (i.e. is not defined in the Investigator Brochure) it will be classified as a Suspected Unexpected Serious Adverse Reaction (SUSAR).

**9.2.3 Reporting to the competent authority and main research ethics committee**

**9.2.3.1 Suspected unexpected serious adverse reactions**

The Trials Office will report a minimal data set of all individual events categorized as a fatal or life threatening SUSAR main Research Ethics Committee (REC) within 7 days. Detailed follow-up information will be provided within an additional 8 days.

All other events categorized as SUSARs will be reported within 15 days.

**9.2.3.2 Serious adverse reactions**

The Trials Office will report details of all SARs (including SUSARs) to main REC annually from the date of the Clinical Trial Authorization, in the form of an Annual Safety Report.

**9.2.3.3 Adverse events**

Details of all AEs will be reported to the local authority on request.

**9.2.3.4 Other safety issues identified during the course of the trial**

The local authority and main REC will be notified immediately if a significant safety issue is identified during the course of the trial.

**9.2.4 Investigators**

Details of all SUSARs and any other safety issue which arises during the course of the trial will be reported to Principal Investigators. A copy of any such correspondence should be filed in the ISF.

**9.2.5 Data monitoring committee**

The independent Data Monitoring Committee (DMC) will review all SAEs.

**9.2.6 Manufacturer of advance therapy medicinal product**

All SAEs will be reported to the manufacturer of the Advanced Therapy Investigational Medicinal Product within 24 hours by fax.

**10. Data handling and record keeping**

**10.1 Data collection**

The CRF must be completed, signed/dated and returned to the CORRECT Trial Office by the Investigator or an authorized member of the site research team (as delegated on the Site Signature and Delegation Log) within the timeframe listed below in Table 1. The exception is the SAE Form, which must be co-signed by the Investigator. See AE reporting Section 8.2 for further details.

Table 1: The Case Report Forms (CRFs)

| Case Report Form | Timelines for completion & return to Trial office |
| --- | --- |
| Baseline Assessment Form | Complete prior to treatment |
| Eligibility Checklist | Complete during screening and return immediately following recruitment of the patient |
| Treatment form | Complete the form at the end of each cycle. |
| Follow-up form | Complete after each treatment cycle |
| Adverse Event Monitoring Form | Complete at baseline (i.e. immediately after recruitment) and on completion of each treatment cycle (as per treatment form) |
| Concomitant Medication Form | Complete at baseline (i.e. immediately after recruitment) and on completion of each treatment cycle (as per treatment form) |
| Treatment Discontinuation Form | Complete when the patient finishes treatment regardless of cause |
| SAE Form | Complete the form within 24h of first awareness of the event and fax to the Trials Office immediately (See section 8.2) |
| Relapse Form | Complete immediately upon patient relapse |
| Death Form | Complete immediately upon notification of patient’s death |
| Pregnancy Notification Form | Complete immediately on being notified of patient pregnancy |
| Deviation Form | Complete immediately upon discovering deviation |
| Withdrawal Form | Complete immediately upon patient withdrawal |

Entries on the CRF should be made in ballpoint pen, in blue or black ink, and must be legible. Any errors should be crossed out with a single stroke, the correction inserted and the change initialed and dated. If it is not obvious why a change has been made, an explanation should be written next to the change.

Data reported on each form should be consistent with the source data or the discrepancies should be explained. If information is not known, this must be clearly indicated on the form. All missing and ambiguous data will be queried. All sections are to be completed before returning to the CORRECT Trial Office.

In all cases it remains the responsibility of the Investigator to ensure that the CRF has been completed correctly and that the data are accurate. The completed originals should be sent to the CORRECT Trials Office and a copy filed in the Investigator Site File.

Trial forms may be amended by the Trials Office, as appropriate, throughout the duration of the trial. Whilst this will not constitute a protocol amendment, new versions of the form must be implemented by participating sites immediately on receipt.

**10.2 Archiving**

It is the responsibility of the Principal Investigator to ensure all essential trial documentation and source records (e.g. signed Informed Consent Forms, Investigator Site Files, Pharmacy Files, patients’ hospital notes, copies of CRFs etc.) at their site are securely retained for at least 5 years after the end of the trial. Participating sites will be sent a letter specifying the permissible disposal date. Do not destroy any documents without prior approval from the CTU.

**11. Quality management**

The trial is being managed under the auspices of the CTU according to the current guidelines for GCP and according to their local procedures. Participating sites will be monitored by CTU staff to confirm compliance with the protocol, and the protection of patients’ rights as detailed in the Declaration of Helsinki (Appendix 6).

**11.1 Site set-up and initiation**

All sites will be required to sign a Clinical Study Site Agreement prior to participation. In addition, all participating Investigators will be asked to sign the necessary agreements, CORRECT trial registration forms, and supply a current CV to the Trials Office. All members of the site research team will also be required to sign the Site Signature and Delegation Log, which should be returned to the Trials Office. Prior to commencing recruitment all sites will undergo a process of initiation. Key members of the site research team will be required to attend a meeting covering aspects of the trial design, protocol procedures, Adverse Event reporting, collection and reporting of data and record keeping. Sites will be provided with an Investigator Site File containing essential documentation, instructions, and other documentation required for the conduct of the trial. The Trials Office must be informed immediately of any change in the site research team.

**11.2 On-site monitoring**

Monitoring will be carried out as required following a risk assessment and as documented in the CORRECT Quality Management Plan. Additional on-site monitoring visits may be triggered for example by poor CRF return, poor data quality, low SAE reporting rates, excessive number of patient withdrawals or deviations. If a monitoring visit is required the Trials Office will contact the site to arrange a date for the proposed visit and will provide the site with written confirmation. Investigators will allow the CORRECT trial staff access to source documents as requested.

**11.3 Central monitoring**

Trials staff will be in regular contact with the site research team to check on progress and address any queries that they may have. Trials staff will check incoming Case Report Forms for compliance with the protocol, data consistency, missing data and timing. Where a patient has given explicit consent sites are requested to send in copies of signed Informed Consent Forms for in-house review. Sites will be sent Data Clarification Forms requesting missing data or clarification of inconsistencies or discrepancies.

Sites may be suspended from further recruitment in the event of serious and persistent non-compliance with the protocol and/or GCP, and/or poor recruitment. Any major problems identified during monitoring may be reported to Trial Management Group and the relevant regulatory bodies. This includes reporting serious breaches of GCP and/or the trial protocol to the main Research Ethics Committee (REC).

**11.4 Audit and inspection**

The investigator will permit trial-related monitoring, audits, ethical review, and regulatory inspection(s) at their site, providing direct access to source data/documents.

**12. End of trial definition**

The end of trial will be 6 months after the last data capture. This will allow sufficient time for the completion of protocol procedures, data collection and data input. The Trials Office will notify main REC that the trial has ended and will provide them with a summary of the clinical trial report within 12 months of the end of trial.

**13. Statistical considerations**

**13.1 Definition of endpoints**

See section 2.2-2.4.

**13.2 Analysis populations**

Efficacy will be assessed in all patients who have received at least one dose of camrelizumab and have an adequate baseline tumor assessment.

Safety analysis will be assessed in all patients who have received at least one dose of camrelizumab.

**13.3 Statistical methods**

PFS and OS will be analyzed by the Kaplan-Meier method for all patients, and compared by the log-rank test for different TMB, PD-L1 and microsatellite status subgroups.

Adverse events will be summarized based on frequency and proportion of all patients. The summaries with descriptive statistics will be given for AEs by any toxicity grade, grade 3 or higher AEs, SAEs and AEs leading to discontinuation of study treatment.

**13.4** **Planned interim analysis**

The primary and secondary outcomes will be reported descriptively and presented to an independent DMC annually, along with information relating to trial recruitment and conduct, data completeness, treatment compliance and safety. Analyses that contribute to DMC reports will include no significance testing unless explicitly requested by DMC.

**13.5 Planned final analysis**

The study will complete recruitment within 2 years and the analysis will take place when all patients have been treated and followed-up for at least 1 year.

**13.6 Sample size calculation**

The sample size calculation is based on the primary outcome of PFS. The two-sides Type I error rate is set at 5% and Type II error rate set at 20%, giving 80% power. The accrual period will be 2 years and follow-up period will be 1 year. The 1-year PFS based upon previous study (7), is 20% among unresectable iCCA patients after gemcitabine plus cisplatin therapy. If these patients receive radiotherapy plus anti-PD-1 therapy, the 1-year PFS will be expected to increase to 40%. It is estimated that 36 patients are required allowing for a 10% loss to follow-up/non-adherence rate.

**14. Trial organizational structure**

**14.1 Sponsor**

The First Affiliated Hospital, Sun Yat-sen University is the legal sponsor of the trial.

**14.2 Coordinating centre**

The trial is being conducted under the auspices of the Clinical Trials Unit (CTU), The First Affiliated Hospital, Sun Yat-sen University according to their local procedures.

**14.3 Trial management group**

A TMG will be established, and will include the Chief Investigator, Local Principal Investigator and other identified collaborators, the Trial Statistician and the Trial Co-ordinator. Key trial personnel will be invited to join the TMG as appropriate to ensure representation from a range of sites and professional groups. The TMG will be responsible for the day-to-day running and management of the trial and will meet by teleconference or in-person as required.

**14.4 Data monitoring committee**

Data analyses will be supplied in confidence to an independent Data Monitoring Committee (DMC), which will be asked to give advice on whether the accumulated data from the trial, together with the results from other relevant researches, justifies the continuing recruitment of further patients. The DMC will operate in accordance with a trial specific charter based upon the template created by the Damocles Group(27). During the recruitment phase of the trial the DMC will meet annually after the trial opens and then one year thereafter until the end of the trial. Additional meetings may be called if recruitment is much faster than anticipated and the DMC may, at their discretion, request to meet more frequently or continue to meet following completion of recruitment. An emergency meeting may also be convened if a safety issue is identified. The DMC will report directly to the Trial Steering Committee. The DMC may consider recommending the discontinuation of the trial if the recruitment rate or data quality are unacceptable or if any issues are identified which may compromise patient safety.

**14.5 Finance**

This is a clinician-initiated and clinician-led trial funded by The First Affiliated Hospital, Sun Yat-sen University. Hengrui enterprise will supply the camrelizumab at no cost for trial patients and fund the CORRECT Trial Office in pursuance of its delegated Sponsor responsibilities.

**15. Ethical considerations**

The trial will be performed in accordance with the recommendations guiding physicians in biomedical research involving human subjects, adopted by the 18th World Medical Association General Assembly, Helsinki, Finland, June 1964, amended at the 48th World Medical Association General Assembly, Somerset West, Republic of South Africa, October 1996 (website: http://www.wma.net/en/30publications/10policies/b3/index.html).

The trial will be conducted in accordance with the International Conference on Harmonization Guidelines for Good Clinical Practice (ICH GCP) and Chinese regulations on advanced therapy. This trial will be carried out in accordance with the related regulations on advanced therapy of Guangdong Province. The protocol will be submitted to and approved by the main Research Ethics Committee (REC) prior to circulation. The protocol will also be approved by the REC of the other participating hospitals before any patients are enrolled into the trial.

It is the responsibility of the Principal Investigator to ensure that all subsequent amendments gain the necessary local approval. This does not affect the individual clinicians’ responsibility to take immediate action if thought necessary to protect the health and interest of individual patients.

**16. Confidentiality and data protection**

Personal data recorded on all documents will be regarded as strictly confidential and will be handled and stored in accordance with the Personal Data Protection Law (exposure draft). With the patient’s consent, their specify patient identifiers e.g. full name, date of birth, address, phone number and hospital number will be collected at trial entry to allow tracing through South China Liver Cancer Registries and to assist with long-term follow-up. Patients will be identified using only their unique trial number, initials (Pinyin), hospital number and date of birth on the Case Report Form and correspondence between the Trials Office and the participating sites. For trials collecting consent forms specify “However patients are asked to give permission for the Trials Office to be sent a copy of their signed Informed Consent Form which will not be anonymized. This will be used to perform in-house monitoring of the consent process”.

The Investigator must maintain documents not for submission to the Trials Office (e.g. Patient Identification Logs) in strict confidence. In the case of specific issues and/or queries from the regulatory authorities, it will be necessary to have access to the complete trial records, provided that patient confidentiality is protected.

The Trials Office will maintain the confidentiality of all patient’s data and will not disclose information by which patients may be identified to any third party other than those directly involved in the treatment of the patient and organisations for which the patient has given explicit consent for data transfer (e.g. laboratory staff). Representatives of the CORRECT trial team may be required to have access to patient’s notes for quality assurance purposes but patients should be reassured that their confidentiality will be respected at all times.

**17. Insurance and indemnity**

The First Affiliated Hospital, Sun Yat-sen University employees are indemnified by the hospital insurers for negligent harm caused by the design or co-ordination of the clinical trials they undertake whilst in the hospital’s employment.

In terms of liability at a site, hospitals have a duty to care for patients treated, whether the patient is taking part in a clinical trial or not. Compensation is therefore cover by the corresponding hospital insurance in the event of clinical negligence having been proven.

The First Affiliated Hospital, Sun Yat-sen University cannot offer indemnity for non-negligent harm.

**18. Publication policy**

Results of this trial will be submitted for publication in a peer-reviewed journal. The manuscript will be prepared by the Trial Management Group (TMG) and authorship will be determined by mutual agreement.

Any secondary publications and presentations prepared by Investigators must be reviewed by the TMG. Manuscripts must be submitted to the TMG in a timely fashion and in advance of being submitted for publication, to allow time for review and resolution of any outstanding issues. Authors must acknowledge that the trial was performed with the support of The First Affiliated Hospital, Sun Yat-sen University. Intellectual property rights will be addressed in the Clinical Study Site Agreement between Sponsor and site.

**19. Reference list**

1. Saha SK, Zhu AX, Fuchs CS, Brooks GA. Forty-Year Trends in Cholangiocarcinoma Incidence in the U.S.: Intrahepatic Disease on the Rise. Oncologist 2016;21:594-599.

2. Khan SA, Taylor-Robinson SD, Toledano MB, Beck A, Elliott P, Thomas HC. Changing international trends in mortality rates for liver, biliary and pancreatic tumours. J Hepatol 2002;37:806-813.

3. Taylor-Robinson SD, Toledano MB, Arora S, Keegan TJ, Hargreaves S, Beck A, Khan SA, et al. Increase in mortality rates from intrahepatic cholangiocarcinoma in England and Wales 1968-1998. Gut 2001;48:816-820.

4. Bergquist A, von Seth E. Epidemiology of cholangiocarcinoma. Best Pract Res Clin Gastroenterol 2015;29:221-232.

5. Bridgewater J, Galle PR, Khan SA, Llovet JM, Park JW, Patel T, Pawlik TM, et al. Guidelines for the diagnosis and management of intrahepatic cholangiocarcinoma. J Hepatol 2014;60:1268-1289.

6. Park SY, Kim JH, Yoon HJ, Lee IS, Yoon HK, Kim KP. Transarterial chemoembolization versus supportive therapy in the palliative treatment of unresectable intrahepatic cholangiocarcinoma. Clin Radiol 2011;66:322-328.

7. Valle J, Wasan H, Palmer DH, Cunningham D, Anthoney A, Maraveyas A, Madhusudan S, et al. Cisplatin plus gemcitabine versus gemcitabine for biliary tract cancer. N Engl J Med 2010;362:1273-1281.

8. Sahai V, Catalano PJ, Zalupski MM, Lubner SJ, Menge MR, Nimeiri HS, Munshi HG, et al. Nab-Paclitaxel and Gemcitabine as First-line Treatment of Advanced or Metastatic Cholangiocarcinoma: A Phase 2 Clinical Trial. JAMA Oncol 2018;4:1707-1712.

9. Shao F, Qi W, Meng FT, Qiu L, Huang Q. Role of palliative radiotherapy in unresectable intrahepatic cholangiocarcinoma: population-based analysis with propensity score matching. Cancer Manag Res 2018;10:1497-1506.

10. Ohkawa A, Mizumoto M, Ishikawa H, Abei M, Fukuda K, Hashimoto T, Sakae T, et al. Proton beam therapy for unresectable intrahepatic cholangiocarcinoma. J Gastroenterol Hepatol 2015;30:957-963.

11. Brunner TB, Blanck O, Lewitzki V, Abbasi-Senger N, Momm F, Riesterer O, Duma MN, et al. Stereotactic body radiotherapy dose and its impact on local control and overall survival of patients for locally advanced intrahepatic and extrahepatic cholangiocarcinoma. Radiotherapy and Oncology 2019;132:42-47.

12. Tao R, Krishnan S, Bhosale PR, Javle MM, Aloia TA, Shroff RT, Kaseb AO, et al. Ablative Radiotherapy Doses Lead to a Substantial Prolongation of Survival in Patients With Inoperable Intrahepatic Cholangiocarcinoma: A Retrospective Dose Response Analysis. J Clin Oncol 2016;34:219-226.

13. Tse RV, Hawkins M, Lockwood G, Kim JJ, Cummings B, Knox J, Sherman M, et al. Phase I Study of Individualized Stereotactic Body Radiotherapy for Hepatocellular Carcinoma and Intrahepatic Cholangiocarcinoma. Journal of Clinical Oncology 2008;26:657-664.

14. Weiner AA, Olsen J, Ma D, Dyk P, DeWees T, Myerson RJ, Parikh P. Stereotactic body radiotherapy for primary hepatic malignancies - Report of a phase I/II institutional study. Radiother Oncol 2016;121:79-85.

15. Hong TS, Wo JY, Yeap BY, Ben-Josef E, McDonnell EI, Blaszkowsky LS, Kwak EL, et al. Multi-Institutional Phase II Study of High-Dose Hypofractionated Proton Beam Therapy in Patients With Localized, Unresectable Hepatocellular Carcinoma and Intrahepatic Cholangiocarcinoma. J Clin Oncol 2016;34:460-468.

16. Boussiotis VA. Molecular and Biochemical Aspects of the PD-1 Checkpoint Pathway. N Engl J Med 2016;375:1767-1778.

17. Iwai Y, Ishida M, Tanaka Y, Okazaki T, Honjo T, Minato N. Involvement of PD-L1 on tumor cells in the escape from host immune system and tumor immunotherapy by PD-L1 blockade. Proc Natl Acad Sci U S A 2002;99:12293-12297.

18. Brahmer JR, Tykodi SS, Chow LQ, Hwu WJ, Topalian SL, Hwu P, Drake CG, et al. Safety and activity of anti-PD-L1 antibody in patients with advanced cancer. N Engl J Med 2012;366:2455-2465.

19. Bardhan K, Anagnostou T, Boussiotis VA. The PD1:PD-L1/2 Pathway from Discovery to Clinical Implementation. Front Immunol 2016;7:550.

20. Piha-Paul SA, Oh DY, Ueno M, Malka D, Chung HC, Nagrial A, Kelley RK, et al. Efficacy and safety of pembrolizumab for the treatment of advanced biliary cancer: Results from the KEYNOTE-158 and KEYNOTE-028 studies. Int J Cancer 2020;147:2190-2198.

21. Ott PA, Bang YJ, Piha-Paul SA, Razak ARA, Bennouna J, Soria JC, Rugo HS, et al. T-Cell-Inflamed Gene-Expression Profile, Programmed Death Ligand 1 Expression, and Tumor Mutational Burden Predict Efficacy in Patients Treated With Pembrolizumab Across 20 Cancers: KEYNOTE-028. J Clin Oncol 2019;37:318-327.

22. Rizvi S, Khan SA, Hallemeier CL, Kelley RK, Gores GJ. Cholangiocarcinoma - evolving concepts and therapeutic strategies. Nat Rev Clin Oncol 2018;15:95-111.

23. Hwang WL, Pike LRG, Royce TJ, Mahal BA, Loeffler JS. Safety of combining radiotherapy with immune-checkpoint inhibition. Nat Rev Clin Oncol 2018;15:477-494.

24. Twyman-Saint Victor C, Rech AJ, Maity A, Rengan R, Pauken KE, Stelekati E, Benci JL, et al. Radiation and dual checkpoint blockade activate non-redundant immune mechanisms in cancer. Nature 2015;520:373-377.

25. Luke JJ, Lemons JM, Karrison TG, Pitroda SP, Melotek JM, Zha Y, Al-Hallaq HA, et al. Safety and Clinical Activity of Pembrolizumab and Multisite Stereotactic Body Radiotherapy in Patients With Advanced Solid Tumors. J Clin Oncol 2018;36:1611-1618.

26. Liniker E, Menzies AM, Kong BY, Cooper A, Ramanujam S, Lo S, Kefford RF, et al. Activity and safety of radiotherapy with anti-PD-1 drug therapy in patients with metastatic melanoma. Oncoimmunology 2016;5:e1214788.

27. A proposed charter for clinical trial data monitoring committees: helping them to do their job well. Lancet 2005;365:711-722.

**Appendix 1 – Diagnosis**

Diagnosis of intrahepatic cholangiocarcinoma is confirmed by biopsy.

**Appendix 2 – Response evaluation criteria in solid tumor**

Measurement of response rate will be based on the RECIST 1.1 Assessment for Hepatocellular Carcinoma. A free copy is available from https://www.ejcancer.com/article/S0959-8049(08)00873-3/fulltext

**Appendix 3** **– ECOG performance status**

| ECOG PERFORMANCE STATUS* | |
| --- | --- |
| Grade | ECOG |
| 0 | Fully active, able to carry on all pre-disease performance without restriction |
| 1 | Restricted in physically strenuous activity but ambulatory and able to carry out work of a light or sedentary nature, e.g., light house work, office work |
| 2 | Ambulatory and capable of all self-care but unable to carry out any work activities. Up and about more than 50% of waking hours |
| 3 | Capable of only limited self-care, confined to bed or chair more than 50% of waking hours |
| 4 | Completely disabled. Cannot carry on any self-care. Totally confined to bed or chair |
| 5 | Dead |

* As published in Am. J. Clin. Oncol.:

Oken MM, Creech RH. Tormey DC, et al. Toxicity And Response Criteria Of The Eastern Cooperative Oncology Group. Am J Clin Oncol 5, 649-655 (1982).

**Appendix 4 – Common terminology criteria for adverse event**

Toxicities will be recorded according to the Common Terminology Criteria for Adverse Events (CTCAE), version 5.0. The full CTCAE document is available on the National Cancer Institute (NCI) website, the following address was correct when this version of the protocol was approved: <http://ctep.cancer.gov/protocolDevelopment/electronic_applications/ctc.htm>

**Appendix 5 – Definition of adverse events**

Adverse Event

Any untoward medical occurrence in a patient or clinical trial subject administered a medicinal product and which does not necessarily have a causal relationship with this treatment.

Comment:

An AE can therefore be any unfavourable and unintended sign (including abnormal laboratory findings), symptom or disease temporally associated with the use of an investigational medicinal product, whether or not related to the investigational medicinal product.

Adverse Reaction

All untoward and unintended responses to an IMP related to any dose administered.

Comment:

An AE judged by either the reporting Investigator or Sponsor as having causal relationship to the IMP qualifies as an AR. The expression reasonable causal relationship means to convey in general that there is evidence or argument to suggest a causal relationship.

Serious Adverse Event

Any untoward medical occurrence or effect that at any dose:

- Results in death
- Is life‑threatening*
- Requires hospitalisation** or prolongation of existing inpatients’ hospitalisation
- Results in persistent or significant disability or incapacity
- Is a congenital anomaly/birth defect
- Or is otherwise considered medically significant by the Investigator***

Comments:

The term severe is often used to describe the intensity (severity) of a specific event. This is not the same as serious, which is based on patients/event outcome or action criteria.

* Life threatening in the definition of an SAE refers to an event in which the patient was at risk of death at the time of the event; it does not refer to an event that hypothetically might have caused death if it were more severe.

**Hospitalisation is defined as an unplanned, formal inpatient admission, even if the hospitalisation is a precautionary measure for continued observation. Thus hospitalisation for protocol treatment (e.g. line insertion), elective procedures (unless brought forward because of worsening symptoms) or for social reasons (e.g. respite care) are not regarded as an SAE.

*** Medical judgment should be exercised in deciding whether an AE is serious in other situations. Important AEs that are not immediately life threatening or do not result in death or hospitalisation but may jeopardise the subject or may require intervention to prevent one of the other outcomes listed in the definition above, should be considered serious.

Serious Adverse Reaction

An Adverse Reaction which also meets the definition of a Serious Adverse Event.

Suspected Unexpected Serious Adverse Reaction

A SAR that is unexpected i.e. the nature, or severity of the event is not consistent with the applicable product information.

A SUSAR should meet the definition of an AR, UAR and SAR.

Unexpected Adverse Reaction

An AR, the nature or severity of which is not consistent with the applicable product information (e.g. Investigator Brochure for an unapproved IMP or (compendium of) Summary of Product Characteristics (SPC) for a licensed product).

When the outcome of an AR is not consistent with the applicable product information the AR should be considered unexpected.

Adverse Event

Any untoward medical occurrence in a patient or clinical trial subject participating in the trial which does not necessarily have a causal relationship with the treatment received.

Comment:

An AE can therefore be any unfavourable and unintended sign (including abnormal laboratory findings), symptom or disease temporally associated with the use of a medicinal product, whether or not related to the medicinal product.

Related Event

An event which resulted from the administration of any of the research procedures.

Serious Adverse Event

An untoward occurrence that:

- Results in death
- Is life-threatening*
- Requires hospitalisation** or prolongation of existing hospitalisation
- Results in persistent or significant disability or incapacity
- Consists of a congenital anomaly/ birth defect
- Or is otherwise considered medically significant by the Investigator***

Comments:

The term severe is often used to describe the intensity (severity) of a specific event. This is not the same as serious, which is based on patients/event outcome or action criteria.

* Life threatening in the definition of an SAE refers to an event in which the patient was at risk of death at the time of the event; it does not refer to an event that hypothetically might have caused death if it were more severe.

**Hospitalisation is defined as an unplanned, formal inpatient admission, even if the hospitalisation is a precautionary measure for continued observation. Thus hospitalisation for protocol treatment (e.g. line insertion), elective procedures (unless brought forward because of worsening symptoms) or for social reasons (e.g. respite care) are not regarded as an SAE.

*** Medical judgment should be exercised in deciding whether an AE is serious in other situations. Important AEs that are not immediately life threatening or do not result in death or hospitalisation but may jeopardise the subject or may require intervention to prevent one of the other outcomes listed in the definition above, should be considered serious.

Unexpected and Related Event

An event which meets the definition of both an Unexpected Event and a Related Event.

Unexpected Event

The type of event that is not listed in the protocol as an expected occurrence.

**Appendix 6 – WMA declaration of helsinki**

World Medical Association Declaration of Helsinki

Recommendations guiding physicians in biomedical research involving human subjects

Adopted by the 18th World Medical Assembly Helsinki, Finland, June 1964 and amended by the 29th World Medical Assembly, Tokyo, Japan, October 1975, 35th World Medical Assembly, Venice, Italy, October 1983, 41st World Medical Assembly, Hong Kong, September 1989 and the 48th General Assembly, Somerset West, Republic of South Africa, October 1996

INTRODUCTION

It is the mission of the physician to safeguard the health of the people. His or her knowledge and conscience are dedicated to the fulfilment of this mission.

The Declaration of Geneva of the WMA binds the physician with the words, "The Health of my patient will be my first consideration," and the International Code of Medical Ethics declares that, "A physician shall act only in the patient's interest when providing medical care which might have the effect of weakening the physical and mental condition of the patient."

The purpose of biomedical research involving human subjects must be to improve diagnostic, therapeutic and prophylactic procedures and the understanding of the aetiology and pathogenesis of disease.

In current medical practice most diagnostic, therapeutic or prophylactic procedures involve hazards. This applies especially to biomedical research.

Medical progress is based on research which ultimately must rest in part on experimentation involving human subjects.

In the field of biomedical research a fundamental distinction must be recognized between medical research in which the aim is essentially diagnostic or therapeutic for a patient, and medical research, the essential object of which is purely scientific and without implying direct diagnostic or therapeutic value to the person subjected to the research.

Special caution must be exercised in the conduct of research which may affect the environment, and the welfare of animals used for research must be respected.

Because it is essential that the results of laboratory experiments be applied to human beings to further scientific knowledge and to help suffering humanity, the WMA has prepared the following recommendations as a guide to every physician in biomedical research involving human subjects. They should be kept under review in the future. It must be stressed that the standards as drafted are only a guide to physicians all over the world. Physicians are not relieved from criminal, civil and ethical responsibilities under the laws of their own countries.

I. BASIC PRINCIPLES

1. Biomedical research involving human subjects must conform to generally accepted scientific principles and should be based on adequately performed laboratory and animal experimentation and on a thorough knowledge of the scientific literature.
2. The design and performance of each experimental procedure involving human subjects should be clearly formulated in an experimental protocol which should be transmitted for consideration, comment and guidance to a specially appointed committee independent of the investigator and the sponsor provided that this independent committee is in conformity with the laws and regulations of the country in which the research experiment is performed.
3. Biomedical research involving human subjects should be conducted only by scientifically qualified persons and under the supervision of a clinically competent medical person. The responsibility for the human subject must always rest with a medically qualified person and never rest on the subject of the research, even though the subject has given his or her consent.
4. Biomedical research involving human subjects cannot legitimately be carried out unless the importance of the objective is in proportion to the inherent risk to the subject.
5. Every biomedical research project involving human subjects should be preceded by careful assessment of predictable risks in comparison with foreseeable benefits to the subject or to others. Concern for the interests of the subject must always prevail over the interests of science and society.
6. The right of the research subject to safeguard his or her integrity must always be respected. Every precaution should be taken to respect the privacy of the subject and to minimize the impact of the study on the subject's physical and mental integrity and on the personality of the subject.
7. Physicians should abstain from engaging in research projects involving human subjects unless they are satisfied that the hazards involved are believed to be predictable. Physicians should cease any investigation if the hazards are found to outweigh the potential benefits.
8. In publication of the results of his or her research, the physician is obliged to preserve the accuracy of the results. Reports of experimentation not in accordance with the principles laid down in this Declaration should not be accepted for publication.
9. In any research on human beings, each potential subject must be adequately informed of the aims, methods, anticipated benefits and potential hazards of the study and the discomfort it may entail. He or she should be informed that he or she is at liberty to abstain from participation in the study and that he or she is free to withdraw his or her consent to participation at any time. The physician should then obtain the subject's freely-given informed consent, preferably in writing.
10. When obtaining informed consent for the research project the physician should be particularly cautious if the subject is in a dependent relationship to him or her or may consent under duress. In that case the informed consent should be obtained by a physician who is not engaged in the investigation and who is completely independent of this official relationship.
11. In case of legal incompetence, informed consent should be obtained from the legal guardian in accordance with national legislation. Where physical or mental incapacity makes it impossible to obtain informed consent, or when the subject is a minor, permission from the responsible relative replaces that of the subject in accordance with national legislation. Whenever the minor child is in fact able to give a consent, the minor's consent must be obtained in addition to the consent of the minor's legal guardian.
12. The research protocol should always contain a statement of the ethical considerations involved and should indicate that the principles enunciated in the present Declaration are complied with.

II. MEDICAL RESEARCH COMBINED WITH PROFESSIONAL CARE

(Clinical Research)

1. In the treatment of the sick person, the physician must be free to use a new diagnostic and therapeutic measure, if in his or her judgement it offers hope of saving life, reestablishing health or alleviating suffering.
2. The potential benefits, hazards and discomfort of a new method should be weighed against the advantages of the best current diagnostic and therapeutic methods.
3. In any medical study, every patient - including those of a control group, if any - should be assured of the best proven diagnostic and therapeutic method. This does not exclude the use of inert placebo in studies where no proven diagnostic or therapeutic method exists.
4. The refusal of the patient to participate in a study must never interfere with the physician-patient relationship.
5. If the physician considers it essential not to obtain informed consent, the specific reasons for this proposal should be stated in the experimental protocol for transmission to the independent committee (I,
6. The physician can combine medical research with professional care, the objective being the acquisition of new medical knowledge, only to the extent that medical research is justified by its potential diagnostic or therapeutic value for the patient.

III. NON-THERAPEUTIC BIOMEDICAL RESEARCH INVOLVING HUMAN SUBJECTS (Non-Clinical Biomedical Research)

1. In the purely scientific application of medical research carried out on a human being, it is the duty of the physician to remain the protector of the life and health of that person on whom biomedical research is being carried out.
2. The subject should be volunteers - either healthy persons or patients for whom the experimental design is not related to the patient's illness.
3. The investigator or the investigating team should discontinue the research if in his/her or their judgement it may, if continued, be harmful to the individual.
4. In research on man, the interest of science and society should never take precedence over considerations related to the wellbeing of the subject.
