# Supplementary material for: Anti-PD-1 antibody in combination with radiotherapy as first-line therapy for unresectable intrahepatic cholangiocarcinoma
Source: BMC Med. 2024 Apr 19;22:165. doi: 10.1186/s12916-024-03381-4 (PMC11027363; doi:10.1186/s12916-024-03381-4)
Supplement: Supplementary file 2 — Additional File 2: Table S1-S6. Table S1 - Treatment exposure. Table S2 - Disease progression. Table S3 - Post-protocol interventions. Table S4 - 1-year PFS rates, ORR, DCR for different TMB, PD-L1 expression and microsatellite instability subgroups. Table S5 - Radiotherapy-related adverse events. Table S6 - Immune-related adverse events. [file 12916_2024_3381_MOESM2_ESM.docx]

**Supplemental Method**

**Inclusion criteria**

The inclusion criteria were as follows: (1) aged between 18 and 75 years old; (2) histopathologically confirmed unresectable primary or postoperative recurrent iCCA without distant metastasis; (3) no previous radiotherapy or systemic therapy; (4) adequate volume of the uninvolved liver (larger than 700 mL); (5) at least one measurable lesion based on Response Evaluation Criteria in Solid Tumors guideline, version 1.1 criteria; (6) Eastern Cooperative Oncology Group performance status score of 0 or 1; (7) adequate hematologic (absolute neutrophil count ≥ 1.5x10^9^/L, hemoglobin concentration ≥ 90 g/L, platelet count ≥ 100 x10^9^/L), hepatic (albumin ≥ 28 g/L, total bilirubin < 1.5 times the upper limit of normal (ULN), alanine aminotransferase and aspartate aminotransferase < 5×ULN) and renal function (serum creatine < 1.5×ULN, creatinine clearance rate ≥ 45 ml/min); (8) life expectancy of at least 12 weeks.

**Exclusion criteria**

The exclusion criteria were as follows: (1) have acute or chronic active hepatitis B or C virus (HBV or HCV), HBV-DNA > 2000 IU/ml or 10^4^ copy/ml; HCV-RNA > 10^3^ copy/ml; both hepatitis B surface antigen and HCV antibody are positive. If the related results become lower than above standards after anti-viral treatment, the patients are qualified for enrollment; (2) have distal or perihilar cholangiocarcinoma, gallbladder cancer or carcinoma of the ampulla of Vater; have metastasis in extrahepatic distant organs including lung, central nervous system, bone etc., or extrahepatic lymph node metastasis beyond abdomen; (3) have risky bleeding events requiring transfusion, operation or local therapies, continuous medication in the past three months; (4) have thromboembolism in the past six months, including myocardial infarction, unstable angina, stroke or transient ischemic attack, pulmonary embolism, deep vein thrombosis; (5) have taken aspirin (> 325 mg/day) or other antiplatelet drugs continuously for more than 10 days within two weeks before enrollment; (6) uncontrollable hypertension, systolic pressure > 140 mmHg or diastolic pressure > 90 mmHg after best medical care, or history of hypertensive crisis or hypertensive encephalopathy; (7) Symptomatic congestive heart failure (New York Heart Association class II-IV). Symptomatic or badly-controlled arrhythmia. Congenital long QT syndrome or modified QTc > 500 ms upon screening; (8) have active autoimmune diseases that require systemic treatment within two years before enrollment; (9) active tuberculosis, having antituberculosis therapy at present or within one year; (10) have a known history of prior invasive malignancies within five years before enrollment; (11) pregnant or breastfeeding women, or expecting to conceive or father children within the projected duration of the study; (12) have other uncontrollable comorbidities; (13) infection of human immunodeficiency virus, known syphilis requiring treatment; (14) allergic to elements of camrelizumab.**Evaluation of tumor mutational burden (TMB) and microsatellite status**

Tumor biopsy was performed within 7 days before therapy. Total DNA was extracted from the snap-frozen tissues using the QIAGEN DNeasy Blood and Tissue Kit (Qiagen, Hilden, Germany). TMB, considered the number of mutations per megabase (mb) of genome examined, was detected and quantified with whole exome sequencing data with a validated algorithm. Library construction was performed using KAPA Hyper Library Kits and a set of 8 nt UDI barcodes (Bioo scientific NEXTFLEX UDI Barcodes). Libraries were analyzed using comprehensive genomic profiling with hybrid capture-based next-generation sequencing for 639 cancer-related genes and introns from 39 genes frequently rearranged in cancer (panel synthesis by Twist Bioscience). The Illumina NovaSeq 6000 platform was used in 2×150 paired end sequencing, and > 2000X unique coverage was generated for most samples. We divided total non-synonymous mutations by our panel size (~1.5 Mb) to calculate TMB in each sample. Mutations likely or known to be bona fide oncogenic drivers and germline polymorphisms were excluded. To further infer microsatellite instability (MSI) in each sample, MSI sensor was applied to calculate the somatic MSI level by comparing microsatellite distributions in tumors and the matched normal samples. Patients with MSI scores greater than 3.5 and TMB ≥ 10 mutations/Mb were defined as MSI, otherwise, they were regarded as microsatellite stability.

In this study, 2.5 mutations/mega-base (Mb) was chosen as the cutoff value for TMB (average TMB according to The Cancer Genome Atlas-CHOL dataset).1 TMB higher than 2.5 mutations/Mb was considered TMB-high and TMB less than 2.5 mutations/Mb was considered TMB-low.

PD-L1 expression was assessed by 22C3 immunohistochemistry staining. PD-L1 expression on tumor cells as well as immune cells were evaluated. PD-L1 positive status was defined as staining in ≥1% of tumor cells or presence of staining of any intensity in tumor-infiltrating immune cells at any intensity.

1. Mody K, Jain P, El-Refai SM, et al. Clinical, Genomic, and Transcriptomic Data Profiling of Biliary Tract Cancer Reveals Subtype-Specific Immune Signatures. JCO Precis Oncol. Jun 2022;6:e2100510. doi:10.1200/PO.21.00510.

**Table S1.** Treatment exposure.

|  | **Patients (n=36)** |
| --- | --- |
| **Radiotherapy dose, n (%)** |  |
| 55 Gy in 25 fractions | 8 (22.2) |
| 50 Gy in 25 fractions | 15 (41.7) |
| 50 Gy in 20 fractions | 3 (8.3) |
| 45 Gy in 20 fractions | 10 (27.8) |
| **Number of cycles of** **Camrelizumab, median (range)** | 15 (2-31) |
| 2 | 2 (5.6) |
| 3 | 3 (8.3) |
| 4 | 1 (2.8) |
| 5 | 1 (2.8) |
| 7 | 3 (8.3) |
| 8 | 1 (2.8) |
| 9 | 3 (8.3) |
| 11 | 1 (2.8) |
| 13 | 2 (5.6) |
| 15 | 7 (19.4) |
| 16 | 4 (11.1) |
| 17 | 2 (5.6) |
| 23 | 3 (8.3) |
| 27 | 1 (2.8) |
| 29 | 1 (2.8) |
| 31 | 1 (2.8) |
| Data are n (%). |  |

| **Table S2.** Disease progression. | |
| --- | --- |
| **Variables** | **Patients (n=20)** |
| Local progression | 1 (5.0) |
| Local + intrahepatic progression | 2 (10.0) |
| Intrahepatic progression | 6 (30.0) |
| Distant progression | 2 (10.0) |
| Intrahepatic progression + distant progression | 9 (45.0) |
| Data are n (%). | |

| **Table S3.** Post-protocol interventions. | |
| --- | --- |
| **Variables** | **Patients (n=21)** |
| Chemotherapy | 18 (85.7) |
| Best supportive care | 3 (14.3) |
| Data are n (%). | |

**Table S4.** 1-year PFS rates, ORR, DCR for different TMB, PD-L1 expression and microsatellite instability subgroups.

|  | **1-year PFS rate** | **ORR** | **DCR** |
| --- | --- | --- | --- |
| **TMB** |  |  |  |
| Low (n=21) | 33.3 (18.2-61.0) | 9 (42.9) | 16 (76.2) |
| High (n=15) | 60.0 (39.7-90.7) | 13 (86.7) | 15 (100.0) |
| **PD-L1 expression** |  |  |  |
| Negative (n=30) | 43.3 (28.8-65.2) | 18 (60.0) | 25 (83.3) |
| Positive (n=6) | 50.0 (22.5-100.0) | 4 (66.7) | 6 (100.0) |
| **Microsatellite status** |  |  |  |
| MSS (n=32) | 40.6 (26.7-61.8) | 19 (59.4) | 27 (84.4) |
| MSI (n=4) | 75.0 (42.6-100.0) | 3 (75.0) | 4 (100.0) |
| Data are n (%) or n (% [95% CI]), unless specified otherwise.  CI, confidence interval; DCR, disease control rate; PD-L1, programmed death-ligand 1; PFS, progression-free survival; MSI, microsatellite instability; MSS, microsatellite stability; ORR, objective response rate; TMB, tumor mutational burden. | | | |

**Table S5.** Radiotherapy-related adverse events.

|  | **All grade incidence (%)** | **Grade≥3 incidence (%)** |
| --- | --- | --- |
| **Hematologic toxic effects** |  |  |
| Decreased lymphocyte | 8 (22.2) | 2 (5.6) |
| Decreased white-cell count | 7 (19.4) | 0 |
| Decreased neutrophils | 5 (13.9) | 0 |
| Anemia | 1 (2.8) | 0 |
| Decreased platelet count | 0 | 0 |
| **Liver function** |  |  |
| Increased ALT | 2 (5.6) | 0 |
| Increased AST | 1 (2.8) | 0 |
| Hypoalbuminemia | 1 (2.8) | 0 |
| **Nonhematologic toxic effects** |  |  |
| Decreased appetite | 3 (8.3) | 0 |
| Abdominal pain | 3 (8.3) | 0 |
| Biliary tract infection | 3 (8.3) | 0 |
| Fatigue | 2 (5.6) | 0 |
| Nausea | 2 (5.6) | 0 |
| Vomiting | 2 (5.6) | 0 |
| Diarrhea | 1 (2.8) | 0 |
| Rash | 1 (2.8) | 0 |
| Fever | 1 (2.8) | 0 |
| Deep-vein thrombosis | 1 (2.8) | 1 (2.8) |
| Reactive cutaneous capillary endothelial proliferation | 0 | 0 |
| Proteinuria | 0 | 0 |
| Hypothyroidism | 0 | 0 |
| Data are presented as n (%). Adverse events were graded according to Common Terminology Criteria for Adverse Events version 5.0.  ALT, alanine aminotransferase; AST, aspartate aminotransferase. | | |

**Table S6.** Immune-related adverse events.

|  | **All grade incidence (%)** | **Grade≥3 incidence (%)** |
| --- | --- | --- |
| **Hematologic toxic effects** |  |  |
| Decreased platelet count | 1 (2.8) | 1 (2.8) |
| Decreased white-cell count | 1 (2.8) | 0 |
| Decreased neutrophils | 1 (2.8) | 0 |
| Anemia | 1 (2.8) | 0 |
| Decreased lymphocyte | 0 | 0 |
| **Liver function** |  |  |
| Increased ALT | 2 (5.6) | 0 |
| Increased AST | 2 (5.6) | 0 |
| Hypoalbuminemia | 0 | 0 |
| **Nonhematologic toxic effects** |  |  |
| Reactive cutaneous capillary endothelial proliferation | 9 (25.0) | 0 |
| Rash | 4 (11.1) | 0 |
| Fatigue | 3 (8.3) | 0 |
| Fever | 3 (8.3) | 0 |
| Nausea | 2 (5.6) | 0 |
| Proteinuria | 2 (5.6) | 0 |
| Hypothyroidism | 2 (5.6) | 0 |
| Decreased appetite | 2 (5.6) | 0 |
| Abdominal pain | 2 (5.6) | 0 |
| Bullous dermatitis | 1 (2.8) | 1 (2.8) |
| Diarrhea | 1 (2.8) | 0 |
| Deep-vein thrombosis | 0 | 0 |
| Biliary tract infection | 0 | 0 |
| Vomiting | 0 | 0 |
| Data are presented as n (%). Adverse events were graded according to Common Terminology Criteria for Adverse Events version 5.0.  ALT, alanine aminotransferase; AST, aspartate aminotransferase. | | |
